# The spatial patterns of diversity and their relationships with environments in rhizosphere microorganisms and host plants differ along elevational gradients

**DOI:** 10.3389/fmicb.2023.1079113

**Published:** 2023-02-23

**Authors:** Shijia Xu, Yan Yuan, Pengfei Song, Mufeng Cui, Rensheng Zhao, Xiaoyang Song, Min Cao, Yazhou Zhang, Jie Yang

**Affiliations:** ^1^CAS Key Laboratory of Tropical Forest Ecology, Xishuangbanna Tropical Botanical Garden, Chinese Academy of Sciences, Mengla, Yunnan, China; ^2^School of Ethnic Medicine, Key Laboratory of Chemistry in Ethnic Medicinal Resources, State Ethnic Affairs Commission and Ministry of Education of China, Yunnan Minzu University, Kunming, Yunnan, China

**Keywords:** microbial diversity, plant diversity, elevational patterns, rhizosphere, biodiversity

## Abstract

**Introduction:**

Identifying spatial patterns of biodiversity along elevational gradients provides a unified framework for understanding these patterns and predicting ecological responses to climate change. Moreover, microorganisms and plants are closely interconnected (e.g., *via* the rhizosphere) and thus may share spatial patterns of diversity and show similar relationships with environments.

**Methods:**

This study compared diversity patterns and relationships with environments in host plants and rhizosphere microorganisms (including various functional groups) along elevational gradients across three climatic zones.

**Results:**

We found that above-and belowground diversity decreased monotonically or showed a hump-shaped or U-shaped pattern along elevation gradients. However, the diversity patterns of plants, bacteria, and fungi varied depending on the taxon and climatic zone. Temperature and humidity strongly contribute to above-and belowground diversity patterns and community composition along elevational gradients. Nonetheless, soil factors might be important regulators of diversity patterns and the community composition of plants and microorganisms along these gradients. Structural equation modeling revealed that environmental factors had a stronger direct effect on rhizosphere microbial diversity than host plant diversity.

**Discussion:**

In sum, spatial patterns of diversity and their relationships with environments in rhizosphere microorganisms and their host plants differed at the regional scale. Different functional groups (e.g., pathogen, mycorrhiza and nitrifier) of soil microorganisms may have divergent elevational patterns and environmental responses. These data improve our understanding of elevational diversity patterns, and provide new insights into the conservation of biodiversity and ecosystem management, especially under climate change.

## Introduction

Biodiversity, the variety of species and ecosystems, plays essential roles in human survival by providing raw materials (e.g., food, medicine, and wood) and fundamental processes (e.g., climate regulation and flood control; Rands et al., 2010). However, with rapid population growth over the past few decades, humans have massively degraded the environment, leading to a substantial and irreversible loss of biodiversity (Sieck et al., 2011). Therefore, elucidating the temporal–spatial distribution of biodiversity is essential for conservation efforts, ecosystem management, and sustainable development (Hunter and Yonzon, 1993; Hu et al., 2020), especially in biodiversity hotspots (Zhang et al., 2021; Zhang Y. Z. et al., 2022). Current conservation agendas focus on macroorganisms (e.g., animals and plants) but neglect microorganisms, which are the greatest source of biodiversity and have important ecosystem functions and services (Guerra et al., 2021). And microbes are an essential component of the ecosystem response to climate change (Monson et al., 2006; Carney et al., 2007). However, the biogeographical patterns and maintenance processes of microorganisms are less well known than those of macroorganisms because of the small size, large abundance, wide distribution, and rapid reproduction of the former (De Wit and Bouvier, 2006; Ren et al., 2018). Therefore, our limited knowledge of microbial diversity does not fit its crucial roles in ecosystem functions and is inadequate to address the threat of the Anthropocene (e.g., climate change and human disturbance; Bodelier, 2011; Zhou and Ning, 2017; Guerra et al., 2020).

Mountains possess exceptional biodiversity because of large elevational and environmental gradients generated by drastic climate and topographic changes over short geographical distances (Körner et al., 2017). Plant diversity has complex and unique patterns along elevational gradients (Gebrehiwot et al., 2019). However, previous studies have focused on the aboveground biodiversity distribution along elevational gradients, and little is known about whether the macroecological patterns of microorganisms and macroorganisms and the processes governing these patterns are synchronous (Sutherland et al., 2013). Bryant et al. (2008) were the first to study bacterial and plant diversity along an elevation gradient. Other studies have also revealed similarities and differences in the elevational distribution patterns of plant and microbial diversity. For instance, in the tropical climate, plant diversity may decrease with elevation (Jing, 2004; Unger et al., 2010). Bacterial and fungal diversity had a U-shaped and a monotonic decreasing pattern in the tropics, respectively (Shen et al., 2020). In a temperate climate, tree species diversity decreased with increasing elevation (Ohsawa, 1995) or exhibited a mid-elevation peak (i.e., hump-shaped pattern). While bacterial diversity exhibited no distinctive pattern across the elevational gradient (Shen et al., 2013), and fungal diversity showed a unimodal pattern (Miyamoto et al., 2014). In general, plants and microorganisms may show different elevational patterns in a single climatic zone. However, these diversity patterns were explored using different experimental designs in single climatic or limiting data comparison. Thus, underscoring the need to study these patterns across different climatic zones using standardized approaches.

Environmental factors significantly influence biodiversity distribution along elevation gradients (i.e., environmental drivers). For instance, temperature and water availability are the primary drivers of plant distribution on a broad scale. In Eurasia’s mountains, microbial diversity and community structure are best predicted by the mean temperature of the warmest quarter and the mean precipitation of the coldest quarter (Picazo et al., 2020). Additionally, microclimates determined the ecology and development of soil microorganisms along an elevational gradient in a subtropical forest (Ma et al., 2022). Soil characteristics are crucial for the diversity of above-and belowground biota. For instance, the main factor influencing biodiversity and community composition along an elevational gradient in mountainous areas is soil pH, particularly for bacteria (Wang et al., 2014; Liu et al., 2021). This diversity is also affected by soil phosphorus, carbon-to-nitrogen ratio (Li et al., 2016), and potassium ion concentrations (Singh et al., 2013). Nonetheless, little is known about the relative importance of climatic and soil factors in above-and belowground diversity, and whether relationships between diversity and the environment differ between microorganisms and plants. Exploring the environmental responses of biodiversity will promote the understanding of biodiverse maintenance mechanisms and the development of ecosystem managements.

However, the rhizosphere microbiome is widely considered the appurtenance of host plants, i.e., it may be greatly shaped by host plants (Berendsen et al., 2012). Plants interact with soil microorganisms through roots and shape microbial diversity by secreting secondary metabolites (Berendsen et al., 2012; Reinhold-Hurek et al., 2015). Therefore, rhizosphere microorganisms and host plants are expected to share spatial patterns of diversity and show similar diversity-environment relationships. However, there may be no significant relationship between the diversity pattern of mycorrhizal fungi and host plants along latitudinal gradients (e.g., Zheng et al., 2021), suggesting host plant effects on microbial abundance and composition in the rhizosphere are highly specific and variable (Costa et al., 2006; Garbeva et al., 2008; Philippot et al., 2013). Here, we assumed that the spatial patterns of diversity and their relationships with environments in rhizosphere microorganisms and their host plants differed at regional scales.

Moreover, various functional groups of soil microorganisms (e.g., mycorrhizal fungi, nitrifier, nitrogen-fixing bacteria and pathogen) play important roles in driving ecosystem functions and services (e.g., climate regulation, nutrient cycling and soil health; Guerra et al., 2020). The majority of previous studies on elevational patterns, however, concentrated on the total bacteria or fungi (Praeg et al., 2019) or a single functional taxon (e.g., ectomycorrhizal fungi; Gong et al., 2022), rarely on different/various functional taxa. Studying and comparing the biodiversity patterns of different functional groups (especially across climatic zones) can help to enhance our understanding of the strength of soil biodiversity in relation to ecosystem function, and better inform management and policy decisions.

This study analyzed the biodiversity patterns of rhizosphere microorganisms (including various functional groups) and their host plants along elevational gradients in tropical, subtropical, and subalpine ecosystems in Yunnan Province, China, and identified environmental factors that shape biodiversity and community composition. The study addressed the following questions: (1) What are the biodiversity patterns of host plants and rhizosphere microorganisms in various climatic zones? (2) What are the environmental factors that affect the biodiversity and community composition of host plants and rhizosphere microorganisms in various climatic zones?

## Methods and materials

### Study sites

**Figure 1:**
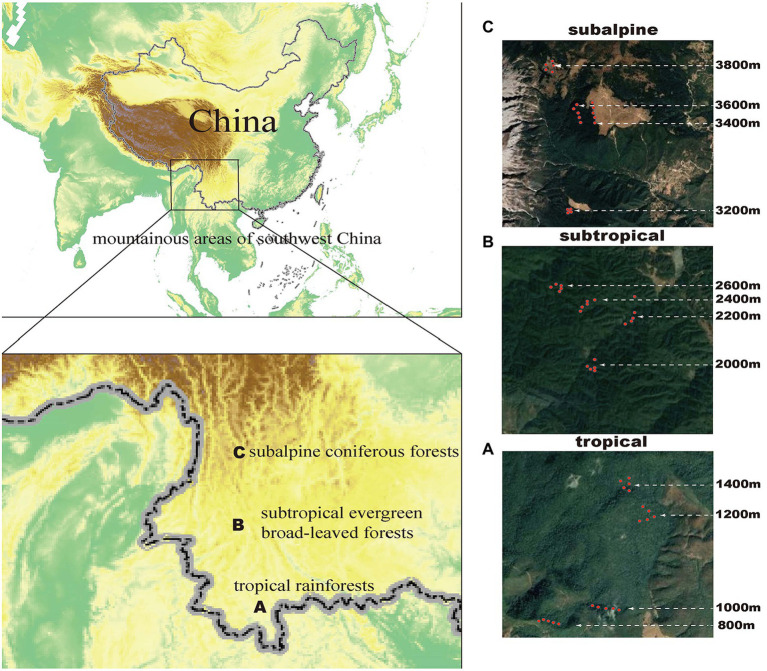
Surveyed sites in Yunnan Province, southwestern China. **(A)** Tropical region (Xishuangbanna Banma Mountain: 800, 1,000, 1,200, and 1,400 m); **(B)** Subtropical region (Puer Ailao Mountain: 2,000, 2,200, 2,400, and 2,600 m); **(C)** Subalpine region (Lijiang Yulong Snow Mountain: 3200, 3,400, 3,600, and 3,800 m). The red dots in **A, B**, and **C** represent the location of each plot.

This study included three sites in Yunnan Province, China–Banma Mountain in Xishuangbanna, Ailao Mountain in Pu′er, and Yulong Snow Mountain in Lijiang–representing a tropical rainforest, a subtropical evergreen broad-leaved forest, and a subalpine coniferous forest, respectively (Figure 1). These sites are biodiversity hotspots characterized by a high diversity of endemic and rare species and have large elevational gradients (Zhang et al., 2021). Additional information on the climate and flora of the study sites is shown in Supporting Information 1. We standardized several experimental conditions in each climatic zone, including four equidistant elevational transects, i.e., 800, 1,000, 1,200, and 1,400 m in tropical mountains; 2000, 2,200, 2,400, and 2,600 m in subtropical mountains; and 3,200, 3,400, 3,600, and 3,800 m in subalpine mountains (Figure 1). Five plots (20 m × 20 m) were set on each elevational transect, totaling 60 plots. Furthermore, we standardized the experimental procedures in each plot to determine tree species composition, collect soil microorganisms, evaluate soil physical and chemical properties, and monitor climatic data.

### Tree species surveys

In the tropical forest, we identified 141 species from 49 families with 1,034 individuals. The four most abundant plant families were Lauraceae (contains 15 species), Moraceae (contains 9 species), Fagaceae (contains 8 species), and Annonaceae (contains 8 species). In the subtropical forest, we identified 74 species from 27 families with 964 individuals. The three most abundant families were Lauraceae (contains 12 species), Fagaceae (contains 9 species), and Theaceae (contains 8 species). In the subalpine forest, we identified 23 species from 10 families with 621 individuals. The three most abundant families were Pinaceae (contains 5 species), Ericaceae (contains 4 species) and Caprifoliaceae (contains 4 species). Individual trees with a diameter at breast height of ≥5 cm in each plot were tagged, identified, and measured. All tree species were identified by professional botanists from the Xishuangbanna Tropical Botanical Garden Herbarium^1^ (Song et al., 2021).

### Soil sample collection and analysis

We collected rhizosphere soil samples from every tree species (host plants) in each plot during the growing season (July to October 2021). First, the tree species in each plot were chosen using survey information. Then, the topsoil layer containing litter and humus was removed, and fine tertiary roots were identified using the root-seeking technique (Laliberté, 2017). Rhizosphere soil (attached to fine roots) was collected using a soft brush. We collected three to five individuals of each tree species and rhizosphere soils from each individual in three random orientations. The soil was sieved through a sieve (2 mm), freeze-dried, and stored at-40°C for molecular analysis.

Rhizosphere soil was collected in each plot for physicochemical analysis. The soil water content was calculated by subtracting the fresh weight from the dry weight (some soils dried in the oven). The remaining samples were air-dried in the shade and transferred to the Central Laboratory of the Public Technology Service Center, Xishuangbanna Tropical Botanical Garden, and the Chinese Academy of Science for measuring organic matter (OM), total carbon (TC), total nitrogen (TN), hydrolysable nitrogen (HN), total phosphorus (TP), total potassium (TK), available potassium (AK), water content, and soil pH. Detailed measurements are shown in Supplementary Tables S1, S2 (Supporting Information).

### Climate data collection

Mean annual temperatures and air humidity were measured using data loggers (iButtonLink LLC, Whitewater, WI, United States). iButton sensors were attached to PVC pipes with ventilation slots to avoid direct solar radiation and were placed in each plot, spaced 1.3 m apart.

### Molecular and bioinformatic analyzes

Genomic DNA samples from rhizosphere soils were extracted using the OMEGA Soil DNA Kit (M5635-02) following the manufacturer’s instructions (OMEGA Bio-Tek, Norcross, GA, United States) and stored at-20°C for further analysis. DNA concentration was measured using a NanoDrop NC2000 spectrophotometer (Thermo Fisher Scientific, Waltham, MA, United States), and DNA quality was assessed by agarose gel electrophoresis. The bacterial 16S rRNA gene V3-V4 region was amplified by PCR using forwards primer 338F (5′-ACTCCTACGGGAGGCAGCA-3′) and reverse primer 806R (5′-GGACTACHVGGGTWTCTAAT-3′; Apprill et al., 2015). The V1 region of the fungal ITS gene was amplified by PCR using a forwards primer (5′-GGAAGTAAAAGTCGTAACAAGG-3′) and a reverse primer (5′-GCTGCGTTCTTCATCGATGC-3′; White et al., 1990). The amplification conditions are shown in the *Supporting Information*. Paired-end Illumina MiSeq sequencing and library preparation were performed by Personal Biotechnology Co., Ltd. (Shanghai, China). Microbiome bioinformatics analysis was carried out using the Quantitative Insights Into Microbial Ecology pipeline (QIIME2) version 2.0 (Bolyen et al., 2019). Primers were removed using the Cutadapt plugin, and raw sequence data were demultiplexed using the Demux plugin (Martin, 2011). The sequences were filtered and trimmed to eliminate short, low-quality, and chimeric reads using the DADA2 plugin (Callahan et al., 2016). A tree was built by FASTTREE2 using nonsinglet amplicon sequence variations (ASVs) compatible with MAFFT (Katoh et al., 2002; Price et al., 2010). All samples were resampled using the QIIME feature table sparse function. Fungal functional groups were extracted from ASVs based on the FUNGuild database (Nguyen et al., 2016), including ectomycorrhiza (ECM), arbuscular mycorrhiza (AM) and fungal pathogen. Bacterial functional groups were extracted from ASVs based on the Functional Annotation of Prokaryotic Taxa (Faprotax) database (Louca et al., 2016), including nitrifier, nitrogen-fixing bacteria (N-fixation) and bacterial pathogen.

Phylogenetic trees for plant species were constructed using Scenario 3 of V. PhyloMaker version 2 (Jin and Qian, 2022). The tree of life contains 74,533 vascular plant species and all families of extant vascular plants and is widely used in ecology (Zhang Y. et al., 2022). Scenario 3 adds species as polytomies within their parental clades and assigns branch lengths using BLADJ algorithm (Branch Length Adjuster, i.e., setting all other branch lengths by placing the nodes evenly between dated nodes, and between dated nodes and terminals; Jin and Qian, 2022). Scientific names were standardized using the plantlist package (Zhang, 2019).

### Statistical analysis

ASV tables were classified according to climatic zones (tropical, subtropical, and subalpine). ASV tables were used to determine alpha diversity and community composition of host plants, fungi (including total fungi, ectomycorrhiza, arbuscular mycorrhiza and pathogen) and bacteria (including total bacteria, nitrifier, nitrogen-fixing bacteria and pathogen). Species richness (SR), Shannon–Wiener diversity index (Shannon), and phylogenetic diversity (PD) were calculated to measure the alpha diversity of plant and soil microorganisms. SR was calculated as the total number of species in a plot (Zhang et al., 2021). Shannon-wiener index is based on relative abundance data, which is affected by both richness and evenness (Shannon, 1948). PD estimates phylogenetic alpha diversity (Faith, 1992), defined as the sum of branch lengths (from the terminal to the base of the phylogeny) of all species in a plot (Zhang et al., 2021). These diversity indexes were calculated using the picante package (Kembel et al., 2010) and vegan package (Oksanen et al., 2013) in R software.

The relationship between alpha diversity and elevation in the three climatic zones was evaluated by linear regression. We compared the Akaike information criterion (AIC) values of simple and multinomial linear regressions and selected models with smaller AIC values for visualization (Supplementary Table S3 in Supplementary material). The models were visualized using the ggplot2 package in R (Wickham, 2016).

The association between alpha diversity and environmental variables (elevation, OM, TC, TN, TK, AK, TP, HN, humidity, temperature, and soil water content) was assessed by Pearson correlation analysis. The importance of environmental variables to diversity was evaluated by random forest. The random forest method accommodates collinear predictors by distributing the relevance of a variable across all variables (Yang et al., 2021). The explanatory power of environmental variables for three alpha diversity metrics was estimated using the linkET package (Huang, 2021) and randomForest package (Liaw and Wiener, 2007) in R software.

Plant and microbial community compositions were ordinated using nonmetric multidimensional scaling (NMDS) with Bray–Curtis dissimilarity matrices using the *metaMDS* function in the Vegan package (Oksanen et al., 2013). The association of species community composition with environmental factors was evaluated by distance-based redundancy analysis (dbRDA). In this analysis, we performed Hellinger transformation on microbial ASV tables. Diversity indices were correlated with environmental variables using the *rdacca.hp* function in R (Lai et al., 2022). Canonical analyzes (RDA, canonical correspondence analysis, and dbRDA) are the best multivariate statistical approaches for investigating explanatory factors for the matrix of response variables. The overall explanatory power of environmental variables can be calculated using R packages; however, it is challenging to accurately calculate the explanatory power of individual variables because of covariance across variables. The *rdacca.hp* function reduces collinearity among environmental factors.

The impact of environmental factors on microbial diversity was assessed using pathway analysis *via* a piecewise structural equation model approach (Grace et al., 2012). First, we performed principal component analysis of environmental factors (climatic and soil properties), plant diversity, and microbial diversity to extract data on the first axis (PC1, explained variance >70%). Then, two models (A and B) were fitted to the data to examine whether the effects of environmental factors on microbial diversity were direct (model A, in which environmental factors directly affected microbial and plant diversity) or indirect (model B, in which environmental factors directly affected plant diversity, which in turn impacted microbial diversity). These analyzes were performed using the FactoMineR package (Lê et al., 2008) and the piecewiseSEM package (Lefcheck, 2015) in R.

All statistical analyzes were conducted in R version 4.1.2 (R Core Team, 2021).

## Results

### Diversity patterns along elevations in different climatic zones

**Figure 2:**
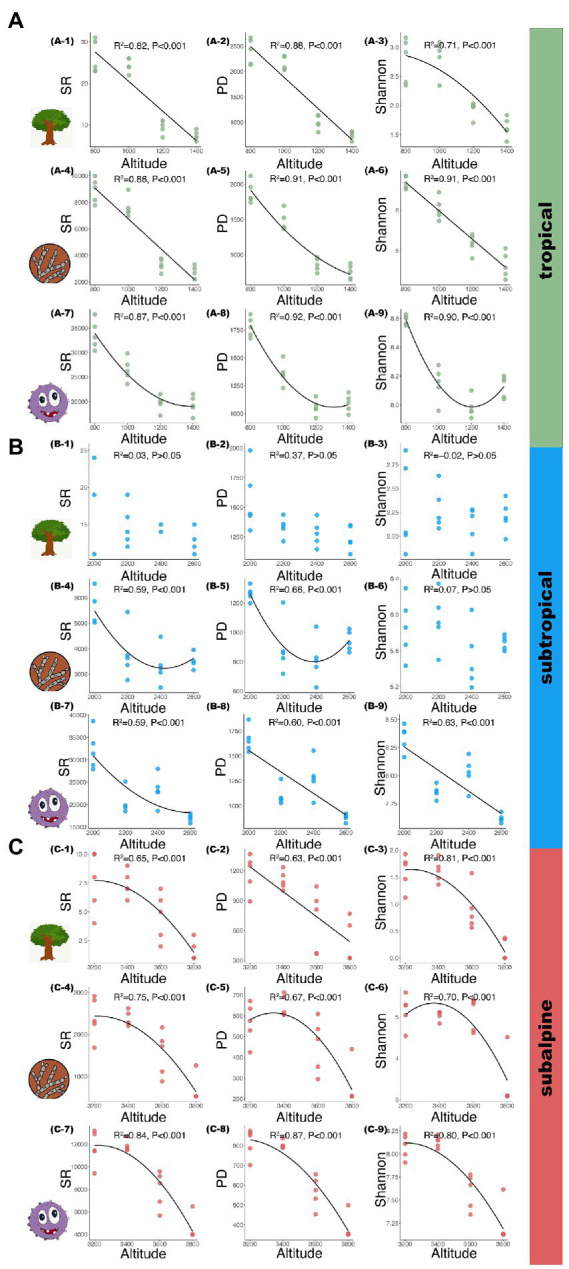
Tree, fungal, and bacterial species along elevational gradients in three climatic zones. **(A)**: tropical; **(B)**: subtropical; **(C)**: subalpine; A (1–3), B (1–3), C (1–3): tree species; A (4–6), B (4–6), C (4–6): fungal species; A (7–9), B (7–9), C (7–9): bacterial species. SR: species richness; PD: phylogenetic diversity; Shannon: Shannon–Wiener index.

The patterns of microbial and plant diversity are shown in Figure 2. Across all soil samples, the dominant phyla of soil fungi were *Basidiomycota*, *Ascomycota*, and *Mortierellomycota*, accounting for more than 80% of the fungal sequences, and they showed increasing, decreasing, and hump-shaped patterns along elevational gradients, respectively; the dominant phyla of soil bacteria were *Proteobacteria*, *Acidobacteria*, *Actinobacteria*, and *Chloroflexi*, accounting for more than 75% of the bacterial sequences, and their distribution patterns across elevational gradient show little variation (Supplementary Figures S1A,B). In the tropical region, the SR, PD, and Shannon index of plants and fungi decreased monotonically with increased elevation [Figure 2A(1–6); *R*^2^: 0.71–0.91, *p* < 0.001]. In contrast, the SR, PD and Shannon index of bacteria showed a U-shaped pattern with increased elevation [Figure 2A(7–9); *R*^2^: 0.87–0.92, *p* < 0.001]. In the subtropical region, the SR, PD and Shannon index of plants had no apparent pattern with increased elevation [Figure 2B(1–3); *p* > 0.05]. For fungal communities, SR and PD exhibited a U-shaped pattern with increased elevation [Figure 2B(4–5); *R*^2^: 0.59–0.66, *p* < 0.001], while Shannon showed no obvious pattern with elevation [Figure 2B-6; *p* > 0.05]. Diversity indexes for bacterial species decreased monotonically with increased elevation [Figure 2B(7–9); *R*^2^: 0.59–0.63, *p* < 0.001]. In the subalpine region, the SR, PD and Shannon index of plants, fungi, and bacteria exhibited a hump-shaped pattern [Figure 2C(1, 3, 4–9); *R*^2^: 0.65–0.87, *p* < 0.001], except for plant PD, which decreased linearly with increasing elevation (Figure 2C; *R*^2^ = 0.63, *p* < 0.001).

The patterns of microbial functional groups diversity are shown in Supplementary Figures S2, S3. In tropical, for fungal functional groups, the alpha diversity of pathogen and AM fungi decreased along increasing elevation (*R*^2^: 0.38–0.98, *p* < 0.001), the ECM diversity showed the opposite trend (Supplementary Figure S2A). For bacterial functional groups (Nitrifier, pathogen and N-fixation), the alpha diversity showed a U-shaped pattern (*R*^2^: 0.38–0.96, *p* < 0.001), except for SR and PD of pathogen, which increased linearly with increased elevation (*R*^2^: 0.6–0.7, *p* < 0.001; Supplementary Figure S3A). In subtropical, fungal functional group diversity show a completely different pattern, including U-shaped patterns for pathogen, decreasing patterns for AM, and hump-shaped or increasing patterns for ECM (Supplementary Figure S2B). Similarly, bacterial functional group diversity show different patterns, including decreasing patterns for Nitrifier, hump-shaped patterns for pathogen and no trend for N_fixation (Supplementary Figure S3B). In subalpine, fungal pathogen and AM diversity showed a decreasing trend along increasing elevation (*R*^2^: 0.23–0.7, *p* < 0.05), while ECM diversity showed an hump-shaped pattern (*R*^2^: 0.47–0.85, *p* < 0.05; Supplementary Figure S2C). Bacterial Nitrifier and pathogen diversity showed a decreasing trend with increased elevation (*R*^2^: 0.28–0.82, *p* < 0.01), while N_fixation diversity showed no significant trend (Supplementary Figure S3C).

### Relationships between environmental factors and diversity across climatic zones

**Figure 3:**
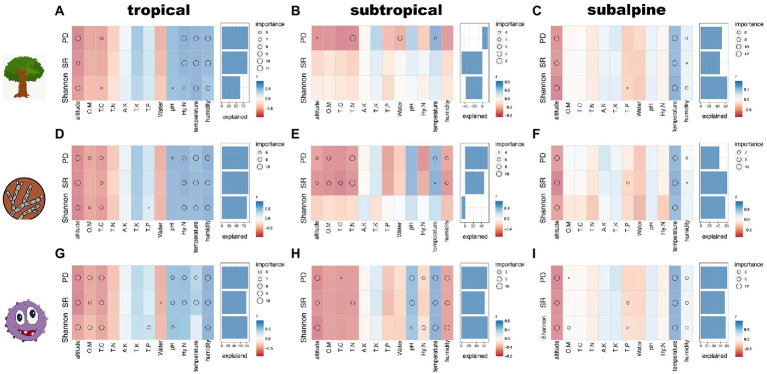
Analysis of environmental factors and diversity indexes in three climatic zones using random forest. **A-C**: tree species; **D-F**: fungal species; **G-I**: bacterial species; OM: soil organic matter content; TC: total carbon; TN: total nitrogen; HN: hydrolysable nitrogen; TP: total phosphorus; TK: total potassium; AK: available potassium; pH: soil pH; water: soil moisture content; temperature: air temperature; humidity: air humidity.

The relationships between diversity and environmental factors fitted by correlation analyzes and random forest models are shown in Figure 3. In the tropical region, predictors explained high SR, PD, and Shannon for host trees (60–80%), fungi (75–85%), and bacteria (80–85%). Soil HN, temperature, and humidity were positively associated with microbial and plant diversity indexes, while elevation had a significant negative effect. Soil pH had a significant and positive effect on bacterial diversity indexes. For soil fungi and bacteria, the correlations between soil nutrients (OM and TC) and diversity indexes were negative (Figures 3A,D,G). In the subtropical region, predictors explained 0–12% of plant diversity, 40–60% of fungal PD and Shannon, and 50–70% of bacterial diversity indexes. Fungal SR and PD were negatively affected by TC, TN, OM, humidity, and elevation positively affected by temperature. Bacterial diversity indexes were negatively associated with elevation and humidity and positively associated with temperature and soil pH (Figures 3B,E,H). In the subalpine region, predictors explained 40–80% of the diversity. Plant, fungal, and bacterial diversity indexes were positively affected by temperature and humidity and negatively affected by elevation (Figures 3C,F,I).

For microbial functional groups, elevation, OM, TC and HN all had a negative effect on their diversity (including fungal pathogen and AM, bacterial Nitrifier and N_fixation) in different climatic zones, while temperature and humidity had a positive effect on them (Supplementary Figure S4). In tropical and subtropical zones, however, these factors have opposite effects on fungal ECM and bacterial pathogen diversity, i.e., elevation, OM, TC and HN have a positive effect, while temperature and humidity have an opposite effect.

### Relationships between environmental factors and community composition across climatic zones

**Figure 4:**
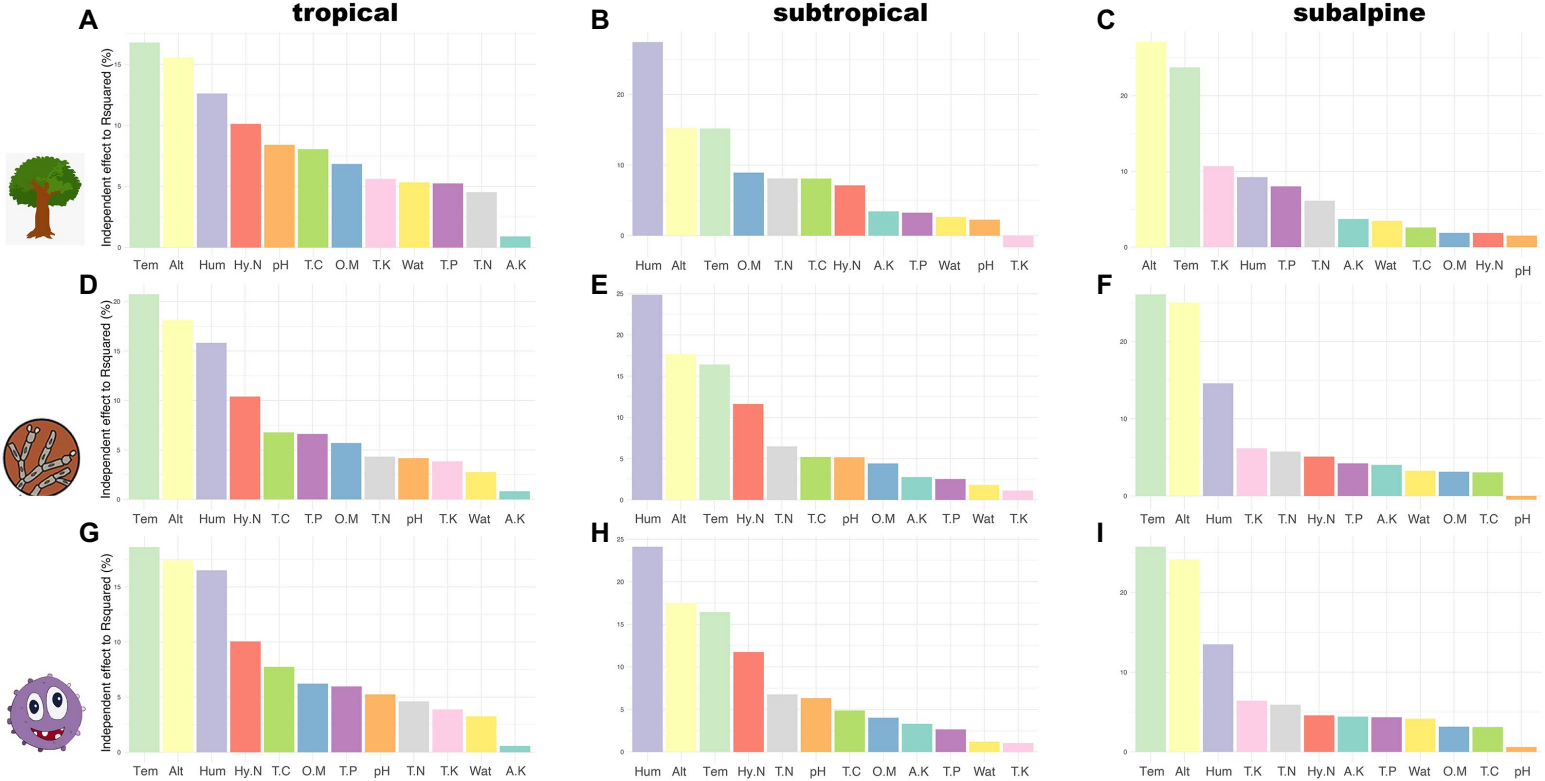
The explanatory power of soil factors for species composition in three climatic zones. **A-C**: tree species; **D-F**: fungal species; **G-I**: bacterial species. OM: organic matter; TC: total carbon; TN: total nitrogen; HN: hydrolysable nitrogen; TP: total phosphorus; TK: total potassium; AK: available potassium; pH: soil pH; Wat: soil moisture content; Alt: altitude/elevation; Tem: air temperature; Hum: air humidity.

The NMDS analysis found that the community composition of host plants and rhizosphere microorganisms differed along elevational gradients and across climatic regions, especially microorganisms, which showed greater differences in community composition than host plants (Supplementary Figure S5). The results of dbRDA for community composition and environmental factors are presented in Figure 4; Supplementary Table S4. In the tropical region, the factors with the highest explanatory power for the community composition of plants, fungi, and bacteria were temperature, elevation, humidity, and HN, and the results were consistent across taxa (Figures 4A,D,G). In the subtropical region, the factors with the highest explanatory power for the community composition of plants, fungi, and bacteria were humidity, elevation, and temperature, and the results were consistent across taxa. However, the fourth predictor was different, i.e., HN had the highest explanatory power for fungi and bacteria, while OM had the highest explanatory power for plants (Figures 4B,E,H). In the subalpine region, the factors with the highest explanatory power (in decreasing order of importance) for tree community composition were elevation, temperature, TK, and humidity, and the factors with the highest explanatory power for fungal and bacterial community composition were temperature, elevation, humidity, and TK (Figures 4C,F,I). In general, the explanatory power of the first four predictive factors (temperature, elevation, humidity, and HN) in the tropical region was 10–20%. The explanatory power of humidity and the remaining variables was 20–30% and 10–20%, respectively, in the subtropical region. The explanatory power of temperature and elevation in the subalpine region was >20%.

**Figure 5:**
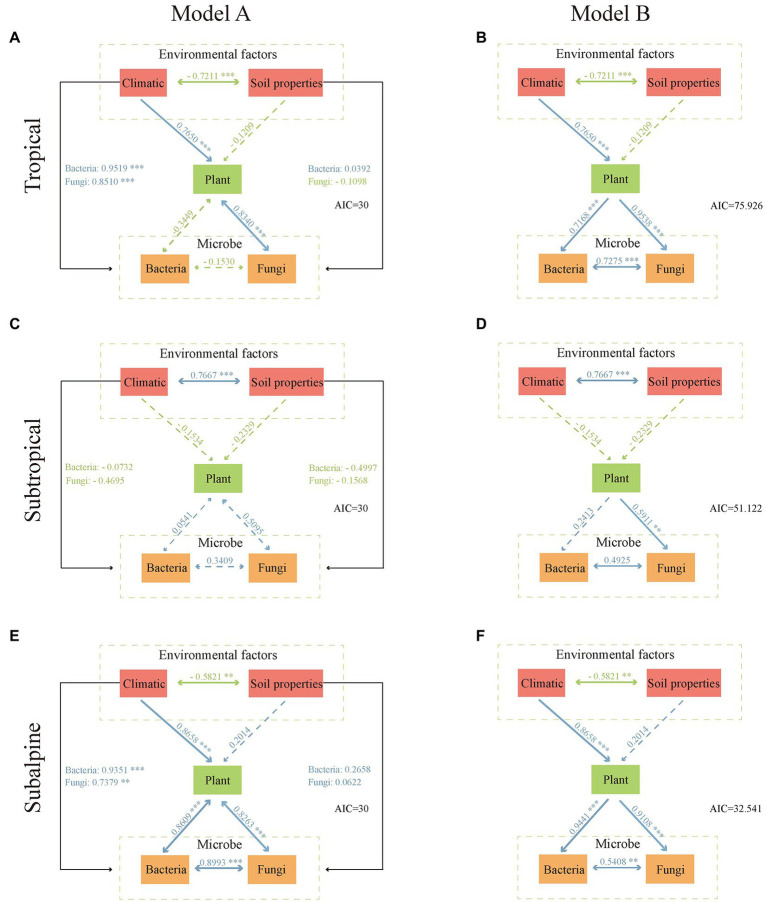
Piecewise structural equation model. We performed principal component analysis of environmental factors (climatic and soil properties), plant diversity, and microbial diversity to extract data on the first axis (PC1, explained variance >70%). Model A: environmental factors directly affect microorganisms and plants. Model B: environmental factors directly influence plant diversity, which in turn affects microorganisms. **(A,B)** tropical; **(C,D)** subtropical; **(E,F)** subalpine. Arrows represent unidirectional or bidirectional relationships among variables. Blue and green arrows denote positive and negative relationships, respectively. Dashed arrows indicate nonsignificant relationships (*p* ≥ 0.05). The thickness of the arrow paths indicates the magnitude of the standardized regression coefficients.

In each climatic zone, model A was better than model B (AIC: 30 vs. 32.5–75.9; Figure 5). Model A indicated that climate significantly affected plant and microbial diversity. In the tropical region, plant diversity was significantly correlated with fungal diversity but not with bacterial diversity. Plant diversity was significantly correlated with fungal and bacterial diversity in the subalpine region but not in the subtropical region. Furthermore, climatic factors had a significantly greater impact on plant and microbial diversity than soil factors.

## Discussion

### Climate-dependent alpha diversity patterns

Consistent with previous studies (Kessler, 2000; Song et al., 2021), we found that the SR, Shannon index, and PD of trees decreased with increasing elevation in the tropical forest (Figure 2). A previous study showed that tree species distribution had a hump-shaped pattern in a subtropical to warm temperate region in the south-eastern part of Nepal (Bhattarai and Vetaas, 2003); however, we found no significant patterns in tree alpha diversity along elevations in the subtropical forest. Except for tree PD, which decreased monotonically, the other diversity indexes in the subalpine forest had a hump-shaped pattern, consistent with the results in Yulong Snow Mountain (Feng et al., 2006) and Meili Snow Mountain (Xin, 2013). These findings demonstrate that plant distribution patterns varied along elevational gradients in different climatic zones.

The distribution patterns of microorganisms varied along elevation in different climatic zones (Figure 2; Supplementary Figures S2, S3). We found that fungal diversity decreased with elevation in the tropical forest, consistent with data from the tropical Andes (Nottingham et al., 2018). Fungal pathogen and AM showed a similar decreased pattern with increasing elevation, while ECM showed an increased pattern. However, bacterial and functional group diversity exhibited a U-shaped pattern (except for pathogen diversity) in the tropics. In the subtropical forest, decreasing patterns of microbial diversity have been reported (Sheng et al., 2019), but U-shaped patterns of fungal SR and PD were rarely reported. In the subalpine forest, we found that the diversity of all analyzed taxa displayed a hump-shaped pattern. Various functional group diversity also demonstrated similarly decreased and hump-shaped patterns.

In conclusion, our analysis indicates that the diversity patterns of plants, bacteria, and fungi (including various functional groups) varied depending on the climatic zone.

### Taxon-dependent alpha diversity patterns

Our research showed that the diversity patterns of fungi and plant were similar (decreased with elevation) in the tropical and subalpine forests (Figure 2). By further analysis, we found that the relative abundance of the second dominant fungi phylum *Ascomycota* decreased with elevation, which is consistent to total fungal patterns (Supplementary Figure S1A). A previous global study found a positive relationship between plant and fungal alpha diversity along a latitudinal gradient, possibly as a result of similar responses to climate and soil factors (Tedersoo et al., 2014). However, the diversity pattern of eukaryotic microbe communities (total and fungal) differed from that of plants in a subalpine mountain (Shen et al., 2014), suggesting that the diversity patterns of fungi and plants may also vary in a single climatic zone.

In addition, the diversity patterns of bacteria were different from those of the other two taxa in the tropical and subtropical regions, in line with a previous study demonstrating that bacteria do not always follow the same biogeographic patterns as plants (Fierer et al., 2011; Wang et al., 2011), which may be because soil bacteria are more susceptible to soil conditions, particularly pH. However, bacterial diversity was closely linked to plant diversity in the subalpine forest, which might be the consequence of cooperation in harsh environments to promote plant growth by solubilizing inorganic and organic element (e.g., phosphorus and nitrogen) pools, and convert them into plant-absorbable forms. Especially, our findings support the above conclusion that bacterial nitrifier diversity greatly affecting nutrient cycling showed similar elevational distribution pattern with host plants in subalpine mountains (Supplementary Figure S3C). In addition, we also found that bacterial and fungal pathogens (associated with soil health; Fierer et al., 2021) had different elevational patterns to other functional groups (nitrifier, N_fixation, ECM, and AM, associated with soil nutrient cycling; Supplementary Figures S3, S4), suggesting that different aspects of ecosystem function and services provided by soils (soil health vs. nutrient cycling) may exhibit inconsistent relationships.

Our findings imply that at large/regional scales, the distribution patterns of rhizosphere microorganisms (including various functional groups) along elevations are not completely synchronous with those of host plants. In addition, the diversity patterns of above-and belowground organisms varied according to taxon and climate. The asynchrony between the diversity patterns of rhizosphere microorganisms and host plants on a large scale may indicate that the influence of host plants on microorganisms is weak at the community level and that microbial diversity is largely controlled by specific responses to the environment. Moreover, the divergent relationships between different functional groups indicate that we should consider the specific differences in different ecosystem functions during policy-making and management processes.

### Shared environmental drivers of alpha diversity and community composition across climatic zones

The diversity patterns and community structures of plants, soil bacteria, and fungi are driven by environmental factors at a large scale (Prober et al., 2015). Nonetheless, few studies have compared drivers of diversity between above-and belowground organisms across climatic zones. Elevation determines species diversity patterns and community composition by integrating many environmental factors, including temperature, humidity, and sunlight (Gaston, 2000), and environmental parameters fluctuate 1,000 times faster along an elevational gradient than along a latitudinal gradient (Tang and Fang, 2004). Our analysis demonstrated that elevation had a considerable negative impact on diversity indices in tropical, subtropical and subalpine zones and that elevation explained 15–20% of the species community composition. Therefore, the effects of elevational gradients on various taxonomic groups were consistent across climatic zones.

Climate is an important driver of diversity along elevations (Heaney, 2001; Lomolino, 2001). We observed that temperature affected the diversity patterns and community structures of different taxa in distinct climatic zones (Figure 3; Supplementary Figure S4). Temperature is fundamental for community development and determines diversity patterns and community composition by regulating metabolism and thermal niches (Janzen, 1967; Colwell et al., 2008; Rapp et al., 2012). We found that temperature was an important predictor of the alpha diversity and community structure of host plant, soil microorganisms and different microbial functional groups, in line with previous studies (Bárcenas-Moreno et al., 2009; Jarvis et al., 2015; Picazo et al., 2020).

The results showed that air humidity greatly influenced plant and microbial (including functional groups) diversity and community composition in all climatic zones (Figures 3, 4; Supplementary Figure S4). Water availability determined the diversity patterns of woody plants along elevations in the Himalayas (Bhattarai and Vetaas, 2006) and the Hengduan Mountains (Liu et al., 2007). Furthermore, water availability is linked to epiphyte diversity (Krömer et al., 2005) and fern diversity (Bhattarai et al., 2004) at different elevations. Water loss affects microbial activity and growth (Bottner, 1985; Kieft et al., 1987) and microbial community structure (Hueso et al., 2012; Sorensen et al., 2013). However, the relative importance of air humidity and soil moisture content in plant and microbial diversity community structures is controversial. For instance, the soil bacterial community structure in the Silver Birch Stand increased with air humidity (Truu et al., 2017). While variations in bacterial abundance in Michigan, United States, were explained by changes in soil moisture content (Buckley and Schmidt, 2001). These results cannot be compared because the studies used different scales and contexts. Our results supported that air humidity has more importance than soil moisture content in determining regional diversity patterns and community compositions. And that climatic conditions consistently determine the diversity patterns and community composition of rhizosphere microorganisms, microbial functional groups and host plants along elevational gradients in different climatic zones. Thus, we can predict the change of above-and below-ground biodiversity in the context of future climate change (especially global warming and abnormal dry) based on their responses to climatic factors in different climatic zones.

### Distinctive environmental drivers of alpha diversity and community composition across climatic zones

The diversity patterns of above-and belowground organisms along elevations are expected to be synchronized because of the consistently dominant effects of climatic factors across climatic regions and taxa. Nonetheless, we observed that diversity patterns differed between rhizosphere microorganisms and host plants, indicating that other factors, e.g., soil conditions, regulate these divergent patterns.

The formation of host plants and rhizosphere bacterial and fungal communities is significantly influenced by soil characteristics (De Ridder-Duine et al., 2005; Andrew et al., 2012). Our findings suggest that the effect of soil factors on diversity patterns and community composition greatly varies depending on taxon and climatic zone (Figures 3, 4). For instance, soil pH significantly impacted bacterial distribution along elevation in different climatic zones. Soil pH may impact soil microorganisms by regulating enzyme activity and nutrient uptake (Wang et al., 2022). Moreover, soil pH predicts mountain biodiversity and community structure, particularly for bacteria (Singh et al., 2012; Shen et al., 2013). Fungal diversity and community structure are less impacted by soil pH than bacterial communities, perhaps because fungal development is optimal in a larger pH range (Rousk et al., 2010). Our results showed that TN had a negative effect on plant, fungal, and bacterial diversity in the subtropical region but not in the other regions. An increase in nitrogen levels may decrease diversity, presumably by increasing litter formation and soil acidity (Fang et al., 2012; Lan and Bai, 2012; Zhang R. et al., 2022). HN increased plant and microbial diversity and community composition in the tropical region. The uptake of HN by plants in tropical forests promotes plant development and diversity, while interactions between plants and microorganisms enhance microbiological richness (Nottingham et al., 2018). Conversely, HN decreased bacterial diversity in the subtropical region. OM and TC were negatively correlated with plant and microbial diversity in tropical and subtropical regions, but there were no obvious relationships in subalpine regions. In addition, for microbial functional groups, EMC and bacterial pathogen have completely different distribution patterns compared to other functional groups, probably due to the opposite effect of the soil factors OM,TC and TN on these two groups (Supplementary Figure S4). In general, diversity patterns are differentially affected by soil factors depending on taxa and climatic regions, e.g., various combinations of significant or nonsignificant effects and positive, negative or neutral relationships.

Our results suggest that environmental factors directly influence rhizosphere microbial diversity (Figure 5), demonstrating that rhizosphere microorganisms are less strongly affected by host plants at large scales. Thus, the differential response of plants, fungi, and bacteria to soil factors reflects the independent effects of environmental factors on host plants and rhizosphere microorganisms at regional scales. Studies suggest that plant diversity dictates rhizosphere microbial diversity at a microscale (Hu et al., 2018; Sasse et al., 2018). However, the spatial patterns of diversity and their relationships with environments in rhizosphere microorganisms and host plants may not be interconnected at regional scales (Fierer et al., 2011; Zheng et al., 2021).

Rather, it seemed that although soil factors have a weaker effect on biodiversity than climate, responses to soil factors vary depending on taxon and climatic zone and account for differences in the diversity patterns of above-and belowground organisms. Further, due to the divergent responses to environmental factors of different functional groups, it is reasonable to make specific management plans, or independently predict ecosystem functions and services in a specific region or taxon.

## Conclusion

This study analyzed the spatial patterns of diversity and their relationships with environments in rhizosphere microorganisms and host plants along elevations in tropical, subtropical, and subalpine forests. These patterns and diversity-environment relationships were asynchronous at large scales. Climate determined the diversity patterns of above-and belowground organisms, while soil properties accounted for the differences in diversity patterns and community compositions. Rhizosphere microbial diversity patterns are controlled by environmental factors other than host plant diversity at regional scales. Thus, the effect of host plants on rhizosphere microbial diversity is weaker at a large scale than at a microscale. Moreover, different functional groups (e.g., pathogen, mycorrhiza and nitrifier) of soil microorganisms may have divergent elevational patterns and environmental responses. These results strengthen our understanding of elevational patterns of soil microorganisms across different climatic zones, improve our knowledge of microbial-plant interactions and help predict the differential response of plants and microorganisms to environmental changes in the context of climate change. Furthermore, these data can contribute to comprehensive ecosystem management in the context of global climate change and biodiversity loss.

## Data availability statement

The data presented in the study are deposited in the National Genomics Data Center, accession number Fungi CRA006600 and Bacteria CRA006619.

## Author contributions

JY and YzZ designed the study. YzZ, SjX, RsZ, MfC, and PfS collected and curated the data. SjX and YzZ analyzed the data. SjX, YY, JY, and YzZ wrote the manuscript.

## Funding

This research was supported by the National Natural Science Foundation of China (32201315 and 31870410), National Key Research and Development Program (2022YFF1302401), China Postdoctoral Science Foundation (2022 M713343), Chinese Academy of Sciences Youth Innovation Promotion Association (Y202080), Distinguished Youth Scholar of Yunnan (202001AV070016), West Light Foundation of the Chinese Academy of Sciences and Ten Thousand Talent Plans for Young Top-Notch Talents of Yunnan Province (YNWR-QNBJ-2018-309), Key Laboratory of Chemistry in Ethnic Medicinal Resources, State Ethnic Affairs Commission and Ministry of Education, Yunnan Minzu University (MY20210507 and MZY2103) and National innovation and entrepreneurship training program for college students (202210691027), and Postdoctoral Fellowship of Xishuangbanna Tropical Botanical Garden, CAS.

## Conflict of interest

The authors declare that the research was conducted in the absence of any commercial or financial relationships that could be construed as a potential conflict of interest.

## Publisher’s note

All claims expressed in this article are solely those of the authors and do not necessarily represent those of their affiliated organizations, or those of the publisher, the editors and the reviewers. Any product that may be evaluated in this article, or claim that may be made by its manufacturer, is not guaranteed or endorsed by the publisher.

## Footnotes

^1^ http://hitbc.Xtbg.ac.cn

## References

[ref1] Andrew D. R. Fitak R. R. Munguia-Vega A. Racolta A. Martinson V. G. Dontsova K. (2012). Abiotic factors shape microbial diversity in Sonoran Desert soils. Appl. Environ. Microb. 78, 7527–7537. doi: 10.1128/AEM.01459-12, PMID: 22885757 PMC3485727

[ref2] Apprill A. McNally S. Parsons R. Weber L. (2015). Minor revision to V4 region SSU rRNA 806R gene primer greatly increases detection of SAR11 bacterioplankton. Aquat. Microb. Ecol. 75, 129–137. doi: 10.3354/ame01753

[ref3] Bárcenas-Moreno G. Gómez-Brandón M. Rousk J. Bååth E. (2009). Adaptation of soil microbial communities to temperature: comparison of fungi and bacteria in a laboratory experiment. Glob. Chang. Biol. 15, 2950–2957. doi: 10.1111/j.1365-2486.2009.01882.x

[ref4] Berendsen R. L. Pieterse C. M. J. Bakker P. A. H. M. (2012). The rhizosphere microbiome and plant health. Trends Plant Sci. 17, 478–486. doi: 10.1016/j.tplants.2012.04.001 22564542

[ref5] Bhattarai K. R. Vetaas O. R. (2003). Variation in plant species richness of different life forms along a subtropical elevation gradient in the Himalayas, East Nepal. Global. Ecol. Biogeogr. 12, 327–340. doi: 10.1046/j.1466-822X.2003.00044.x

[ref6] Bhattarai K. R. Vetaas O. R. (2006). Can Rapoport's rule explain tree species richness along the Himalayan elevation gradient, Nepal? Divers. Distrib. 12, 373–378. doi: 10.1111/j.1366-9516.2006.00244.x

[ref7] Bhattarai K. R. Vetaas O. R. Grytnes J. A. (2004). Fern species richness along a central Himalayan elevational gradient. Nepal. J. Biogeogr. 31, 389–400. doi: 10.1046/j.0305-0270.2003.01013.x

[ref8] Bodelier P. L. (2011). Toward understanding, managing, and protecting microbial ecosystems. Front. Microbiol. 2:80. doi: 10.3389/fmicb.2011.00080 21747797 PMC3128941

[ref9] Bolyen E. Rideout J. R. Dillon M. R. Bokulich N. A. Abnet C. C. Al-Ghalith G. A.. (2019). Reproducible, interactive, scalable and extensible microbiome data science using QIIME 2. Nat. Biotechnol. 37, 852–857. doi: 10.1038/s41587-019-0209-9, PMID: 31341288 PMC7015180

[ref10] Bottner P. (1985). Response of microbial biomass to alternate moist and dry conditions in a soil incubated with 14C-and 15N-labelled plant material. Soil Biol. Biochem. 17, 329–337. doi: 10.1016/0038-0717(85)90070-7

[ref11] Bryant J. A. Lamanna C. Morlon H. Kerkhoff A. J. Enquist B. J. Green J. L. (2008). Colloquium paper: microbes on mountainsides: contrasting elevational patterns of bacterial and plant diversity. Proc. Natl. Acad. Sci. U. S. A. 105, 11505–11511. doi: 10.1073/pnas.0801920105, PMID: 18695215 PMC2556412

[ref12] Buckley D. H. Schmidt T. M. (2001). Environmental factors influencing the distribution of rRNA from Verrucomicrobia in soil. FEMS Microbiol. Ecol. 35, 105–112. doi: 10.1111/j.1574-6941.2001.tb00793.x, PMID: 11248395

[ref13] Callahan B. J. McMurdie P. J. Rosen M. J. Han A. W. Johnson A. J. A. Holmes S. P. (2016). DADA2: high-resolution sample inference from Illumina amplicon data. Nat. Methods 13, 581–583. doi: 10.1038/nmeth.3869, PMID: 27214047 PMC4927377

[ref14] Carney K. M. Hungate B. A. Drake B. G. Megonigal J. P. (2007). Altered soil microbial community at elevated CO_2_ leads to loss of soil carbon. Proc. Natl. Acad. Sci. U. S. A. 104, 4990–4995. doi: 10.1073/pnas.0610045104, PMID: 17360374 PMC1820881

[ref15] Colwell R. K. Brehm G. Cardelús C. L. Gilman A. C. Longino J. T. (2008). Global warming, elevational range shifts, and lowland biotic attrition in the wet tropics. Science 322, 258–261. doi: 10.1126/science.1162547, PMID: 18845754

[ref16] Costa R. Götz M. Mrotzek N. Lottmann J. Berg G. Smalla K. (2006). Effects of site and plant species on rhizosphere community structure as revealed by molecular analysis of microbial guilds. FEMS Microbiol. Ecol. 56, 236–249. doi: 10.1111/j.1574-6941.2005.00026.x, PMID: 16629753

[ref17] De Ridder-Duine A. S. Kowalchuk G. A. Klein Gunnewiek P. J. A. Smant W. van Veen J. A. de Boer W. (2005). Rhizosphere bacterial community composition in natural stands of *Carex arenaria* (sand sedge) is determined by bulk soil community composition. Soil Biol. Biochem. 37, 349–357. doi: 10.1016/j.soilbio.2004.08.005

[ref18] De Wit R. Bouvier T. (2006). 'Everything is everywhere, but, the environment selects'; what did baas Becking and Beijerinck really say? Environ. Microbiol. 8, 755–758. doi: 10.1111/j.1462-2920.2006.01017.x 16584487

[ref19] Faith D. P. (1992). Conservation evaluation and phylogenetic diversity. Biol. Conserv. 61, 1–10. doi: 10.1016/0006-3207(92)91201-3

[ref20] Fang Y. Xun F. Bai W. Zhang W. Li L. (2012). Long-term nitrogen addition leads to loss of species richness due to litter accumulation and soil acidification in a temperate steppe. PLoS One 7:e47369. doi: 10.1371/journal.pone.0047369, PMID: 23077603 PMC3470592

[ref21] Feng J. M. Wang X. Xu C. D. Yang Y. Fang J. Y. (2006). Altitudinal patterns of plant species diversity and community structure on Yulong Mountains, Yunnan, China. Mt. Sci. 24, 110–116.

[ref22] Fierer N. McCain C. M. Meir P. Zimmermann M. Rapp J. M. Silman M. R.. (2011). Microbes do not follow the elevational diversity patterns of plants and animals. Ecology 92, 797–804. doi: 10.1890/10-1170.1 21661542

[ref23] Fierer N. Wood S. A. Bueno de Mesquita C. P. (2021). How microbes can, and cannot, be used to assess soil health. Soil Biol. Biochem. 153:108111. doi: 10.1016/j.soilbio.2020.108111

[ref24] Garbeva P. van Elsas J. Veen J. (2008). Rhizosphere microbial community and its response to plant species and soil history. Plant Soil 302, 19–32. doi: 10.1007/s11104-007-9432-0

[ref25] Gaston K. J. (2000). Global patterns in biodiversity. Nature 405, 220–227. doi: 10.1038/35012228 10821282

[ref26] Gebrehiwot K. Demissew S. Woldu Z. Fekadu M. Desalegn T. Teferi E. (2019). Elevational changes in vascular plants richness, diversity, and distribution pattern in Abune Yosef mountain range, northern Ethiopia. Plant Divers. 41, 220–228. doi: 10.1016/j.pld.2019.06.005, PMID: 31528781 PMC6743012

[ref27] Gong S. Feng B. Jian S. P. Wang G. S. Ge Z. W. Yang Z. L. (2022). Elevation matters more than season in shaping the heterogeneity of soil and root associated ectomycorrhizal fungal community. Microbiol. Spectr. 10:e0195021. doi: 10.1128/spectrum.01950-21, PMID: 35019700 PMC8754124

[ref28] Grace J. B. Schoolmaster D. R. Jr. Guntenspergen G. R. Little A. M. Mitchell B. R. Miller K. M.. (2012). Guidelines for a graph-theoretic implementation of structural equation modeling. Ecosphere 3:art73. doi: 10.1890/ES12-00048.1

[ref29] Guerra C. A. Bardgett R. D. Caon L. Crowther T. W. Delgado-Baquerizo M. Montanarella L.. (2021). Tracking, targeting, and conserving soil biodiversity. Science 371, 239–241. doi: 10.1126/science.abd7926, PMID: 33446546

[ref30] Guerra C. A. Heintz-Buschart A. Sikorski J. Chatzinotas A. Guerrero-Ramirez N. Cesarz S.. (2020). Blind spots in global soil biodiversity and ecosystem function research. Nat. Commun. 11:3870. doi: 10.1038/s41467-020-17688-2, PMID: 32747621 PMC7400591

[ref31] Heaney L. R. (2001). Small mammal diversity along elevational gradients in the Philippines: an assessment of patterns and hypotheses. Glob. Ecol. Biogeogr. 10, 15–39. doi: 10.1046/j.1466-822x.2001.00227.x

[ref32] Hu L. Robert C. A. M. Cadot S. Zhang X. Ye M. Li B.. (2018). Root exudate metabolites drive plant-soil feedbacks on growth and defense by shaping the rhizosphere microbiota. Nat. Commun. 9:2738. doi: 10.1038/s41467-018-05122-7, PMID: 30013066 PMC6048113

[ref33] Hu A. Wang J. Sun H. Niu B. Si G. Wang J.. (2020). Mountain biodiversity and ecosystem functions: interplay between geology and contemporary environments. ISME J. 14, 931–944. doi: 10.1038/s41396-019-0574-x, PMID: 31896789 PMC7082341

[ref34] Huang H.. (2021). *LinkET: everything is Linkable*. R Package Version 0.0.2.9. Available at: https://github.com/Hy4m/linkET.

[ref35] Hueso S. García C. Hernández T. (2012). Severe drought conditions modify the microbial community structure, size and activity in amended and unamended soils. Soil Biol. Biochem. 50, 167–173. doi: 10.1016/j.soilbio.2012.03.026

[ref36] Hunter M. L. Yonzon P. (1993). Altitudinal distributions of birds, mammals, people, forests, and parks in Nepal. Conserv. Biol. 7, 420–423. doi: 10.1046/j.1523-1739.1993.07020420.x

[ref37] Janzen D. H. (1967). Why mountain passes are higher in the tropics. Am. Nat. 101, 233–249. doi: 10.1086/282487

[ref38] Jarvis S. G. Woodward S. Taylor A. F. S. (2015). Strong altitudinal partitioning in the distributions of ectomycorrhizal fungi along a short (300 m) elevation gradient. New Phytol. 206, 1145–1155. doi: 10.1111/nph.13315, PMID: 25655082

[ref39] Jin Y. Qian H. (2022). V.PhyloMaker2: an updated and enlarged R package that can generate very large phylogenies for vascular plants. Plant Divers. 44, 335–339. doi: 10.1016/j.pld.2022.05.005, PMID: 35967255 PMC9363651

[ref40] Jing F.. (2004). *Exploring Altitudinal Patterns of Plant Diversity of China’s Mountains. Chinese Biodiversity*. (In Chinese with English Abstract).

[ref41] Katoh K. Misawa K. Kuma K. Miyata T. (2002). MAFFT: a novel method for rapid multiple sequence alignment based on fast Fourier transform. Nucleic Acids Res. 30, 3059–3066. doi: 10.1093/nar/gkf436, PMID: 12136088 PMC135756

[ref42] Kembel S. W. Cowan P. D. Helmus M. R. Cornwell W. K. Morlon H. Ackerly D. D.. (2010). Picante: R tools for integrating phylogenies and ecology. Bioinformatics 26, 1463–1464. doi: 10.1093/bioinformatics/btq166, PMID: 20395285

[ref43] Kessler M. (2000). Elevational gradients in species richness and endemism of selected plant groups in the central Bolivian Andes. Plant Ecol. 149, 181–193. doi: 10.1023/A:1026500710274

[ref44] Kieft T. L. Soroker E. Firestone M. K. (1987). Microbial biomass response to a rapid increase in water potential when dry soil is wetted. Soil Biol. Biochem. 19, 119–126. doi: 10.1016/0038-0717(87)90070-8

[ref45] Körner C. Jetz W. Paulsen J. Payne D. Rudmann-Maurer K. M. Spehn E. (2017). A global inventory of mountains for bio-geographical applications. Alpine Bot. 127, 1–15. doi: 10.1007/s00035-016-0182-6

[ref46] Krömer T. Kessler M. Robbert Gradstein S. Acebey A. (2005). Diversity patterns of vascular epiphytes along an elevational gradient in the Andes. J. Biogeogr. 32, 1799–1809. doi: 10.1111/j.1365-2699.2005.01318.x

[ref47] Lai J. Zou Y. Zhang J. Peres-Neto P. R. (2022). Generalizing hierarchical and variation partitioning in multiple regression and canonical analyses using the rdacca.Hp R package. Methods Ecol. Evol. 13, 782–788. doi: 10.1111/2041-210X.13800

[ref48] Laliberté E. (2017). Below-ground frontiers in trait-based plant ecology. New Phytol. 213, 1597–1603. doi: 10.1111/nph.14247, PMID: 27735077

[ref49] Lan Z. Bai Y. (2012). Testing mechanisms of N-enrichment-induced species loss in a semiarid Inner Mongolia grassland: critical thresholds and implications for long-term ecosystem responses. Philos. Trans. R. Soc. B Biol. Sci. 367, 3125–3134. doi: 10.1098/rstb.2011.0352, PMID: 23045710 PMC3479690

[ref50] Lê S. Josse J. Husson F. (2008). FactoMineR: an R package for multivariate analysis. J. Stat. Softw. 25:1. doi: 10.18637/jss.v025.i01

[ref51] Lefcheck J. (2015). PIECEWISESEM: piecewise structural equation modelling in R for ecology, evolution, and systematics. Methods Ecol. Evol. 7, 573–579. doi: 10.1111/2041-210X.12512

[ref52] Li G. Xu G. Shen C. Tang Y. Zhang Y. Ma K. (2016). Contrasting elevational diversity patterns for soil bacteria between two ecosystems divided by the treeline. Sci. China Life Sci. 59, 1177–1186. doi: 10.1007/s11427-016-0072-6 27601034

[ref53] Liaw A. Wiener M. C.. (2007). *Classification and Regression by Random Forest*. R News, pp. 18–22. Available at: https://CRAN.R-project.org/doc/Rnews/.

[ref54] Liu Y. Zhang Y. He D. Cao M. Zhu H. (2007). Climatic control of plant species richness along elevation gradients in the longitudinal range-gorge region. Chin. Sci. Bull. 52, 50–58. doi: 10.1007/s11434-007-7006-4

[ref55] Liu L. Zhu K. Krause S. M. B. Li S. Wang X. Zhang Z.. (2021). Changes in assembly processes of soil microbial communities during secondary succession in two subtropical forests. Soil Biol. Biochem. 154:108144. doi: 10.1016/j.soilbio.2021.108144

[ref56] Lomolino M. V. (2001). Elevation gradients of species-density: historical and prospective views. Glob. Ecol. Biogeogr. 10, 3–13. doi: 10.1046/j.1466-822x.2001.00229.x

[ref57] Louca S. Parfrey L. W. Doebeli M. (2016). Decoupling function and taxonomy in the global ocean microbiome. Science 353, 1272–1277. doi: 10.1126/science.aaf4507, PMID: 27634532

[ref58] Ma L. Liu L. Lu Y. Chen L. Zhang Z. Zhang H.. (2022). When microclimates meet soil microbes: temperature controls soil microbial diversity along an elevational gradient in subtropical forests. Soil Biol. Biochem. 166:108566. doi: 10.1016/j.soilbio.2022.108566

[ref59] Martin M. (2011). Cutadapt removes adapter sequences from high-throughput sequencing reads. EMBnet J. 17:200. doi: 10.14806/ej.17.1.200

[ref60] Miyamoto Y. Nakano T. Hattori M. Nara K. (2014). The mid-domain effect in ectomycorrhizal fungi: range overlap along an elevation gradient on Mount Fuji, Japan. ISME J. 8, 1739–1746. doi: 10.1038/ismej.2014.34, PMID: 24621523 PMC4817612

[ref01] Nguyen N. H. Song Z. Bates S. T. Branco S. Tedersoo L. Menke J.. (2016). FUNGuild: An open annotation tool for parsing fungal community datasets by ecological guild. Fungal Ecol. 20, 241–248. doi: 10.1016/j.funeco.2015.06.006

[ref61] Monson R. K. Lipson D. L. Burns S. P. Turnipseed A. A. Delany A. C. Williams M. W.. (2006). Wint by climate and microbial community composition. Nature 439, 711–714. doi: 10.1038/nature04555, PMID: 16467835

[ref62] Nottingham A. T. Fierer N. Turner B. L. Whitaker J. Ostle N. J. McNamara N. P.. (2018). Microbes follow Humboldt: temperature drives plant and soil microbial diversity patterns from the Amazon to the Andes. Ecology 99, 2455–2466. doi: 10.1002/ecy.2482, PMID: 30076592 PMC6850070

[ref63] Ohsawa M. (1995). Latitudinal comparison of altitudinal changes in forest structure, leaf-type, and species richness in humid monsoon Asia. Plant Ecol. 121, 3–10. doi: 10.1007/BF00044667

[ref64] Oksanen J. Blanchet F. G. Kindt R. Legendre P. Minchin P. O’Hara R. B.. (2013). *Vegan: Community Ecology Package*. R Package Version. 2.0-10. Available at: https://CRAN.R-project.org/package=vegan.

[ref65] Philippot L. Raaijmakers J. M. Lemanceau P. van der Putten W. H. (2013). Going back to the roots: the microbial ecology of the rhizosphere. Nat. Rev. Microbiol. 11, 789–799. doi: 10.1038/nrmicro3109, PMID: 24056930

[ref66] Picazo F. Vilmi A. Aalto J. Soininen J. Casamayor E. O. Liu Y.. (2020). Climate mediates continental scale patterns of stream microbial functional diversity. Microbiome 8:92. doi: 10.1186/s40168-020-00873-2 32534595 PMC7293791

[ref67] Praeg N. Pauli H. Illmer P. (2019). Microbial diversity in bulk and Rhizosphere soil of Ranunculus glacialis along a high-alpine altitudinal gradient. Front. Microbiol. 10:1429. doi: 10.3389/fmicb.2019.01429, PMID: 31338073 PMC6629913

[ref68] Price M. Dehal P. Arkin A. (2010). FastTree 2–approximately maximum-likelihood trees for large alignments. PLoS One 5:e9490. doi: 10.1371/journal.pone.0009490 20224823 PMC2835736

[ref69] Prober S. M. Leff J. W. Bates S. T. Borer E. T. Firn J. Harpole W. S.. (2015). Plant diversity predicts beta but not alpha diversity of soil microbes across grasslands worldwide. Ecol. Lett. 18, 85–95. doi: 10.1111/ele.12381 25430889

[ref70] R Core Team. (2021). *R: A Language and Environment for Statistical Computing*. Vienna, Austria: R Foundation for Statistical Computing. Available at: https://www.R-project.org/.

[ref71] Rands M. R. W. Adams W. M. Bennun L. Butchart S. H. M. Clements A. Coomes D.. (2010). Biodiversity conservation: challenges beyond 2010. Science 329, 1298–1303. doi: 10.1126/science.1189138, PMID: 20829476

[ref72] Rapp J. M. Silman M. R. Clark J. S. Girardin C. A. Galiano D. Tito R. (2012). Intra-and interspecific tree growth across a long altitudinal gradient in the Peruvian Andes. Ecology 93, 2061–2072. doi: 10.1890/11-1725.1, PMID: 23094378

[ref73] Reinhold-Hurek B. Bünger W. Burbano C. S. Sabale M. Hurek T. (2015). Roots shaping their microbiome: global hotspots for microbial activity. Annu. Rev. Phytopathol. 53, 403–424. doi: 10.1146/annurev-phyto-082712-102342, PMID: 26243728

[ref74] Ren C. Zhang W. Zhong Z. Han X. Yang G. Feng Y.. (2018). Differential responses of soil microbial biomass, diversity, and compositions to altitudinal gradients depend on plant and soil characteristics. Sci. Total Environ. 610-611, 750–758. doi: 10.1016/j.scitotenv.2017.08.110, PMID: 28822942

[ref75] Rousk J. Bååth E. Brookes P. C. Lauber C. L. Lozupone C. Caporaso J. G.. (2010). Soil bacterial and fungal communities across a pH gradient in an arable soil. ISME J. 4, 1340–1351. doi: 10.1038/ismej.2010.58, PMID: 20445636

[ref76] Sasse J. Martinoia E. Northen T. (2018). Feed your friends: do Plant exudates shape the root microbiome? Trends Plant Sci. 23, 25–41. doi: 10.1016/j.tplants.2017.09.003, PMID: 29050989

[ref77] Shannon C. E. (1948). A mathematical theory of communication. Bell Syst. Tech. J. 27, 623–656. doi: 10.1002/j.1538-7305.1948.tb00917.x

[ref78] Shen C. Gunina A. Luo Y. Wang J. He J. Z. Kuzyakov Y.. (2020). Contrasting patterns and drivers of soil bacterial and fungal diversity across a mountain gradient. Environ. Microbiol. 22, 3287–3301. doi: 10.1111/1462-2920.15090, PMID: 32436332

[ref79] Shen C. Liang W. Shi Y. Lin X. Zhang H. Wu X.. (2014). Contrasting elevational diversity patterns between eukaryotic soil microbes and plants. Ecology 95, 3190–3202. doi: 10.1890/14-0310.1

[ref80] Shen C. Xiong J. Zhang H. Feng Y. Lin X. Li X.. (2013). Soil pH drives the spatial distribution of bacterial communities along elevation on Changbai Mountain. Soil Biol. Biochem. 57, 204–211. doi: 10.1016/j.soilbio.2012.07.013

[ref81] Sheng Y. Cong W. Yang L. Liu Q. Zhang Y. (2019). Forest soil fungal community Elevational distribution pattern and their ecological assembly processes. Front. Microbiol. 10:2226. doi: 10.3389/fmicb.2019.02226 31636612 PMC6787267

[ref82] Sieck M. Ibisch P. L. Moloney K. A. Jeltsch F. (2011). Current models broadly neglect specific needs of biodiversity conservation in protected areas under climate change. BMC Ecol. 11:12. doi: 10.1186/1472-6785-11-12, PMID: 21539736 PMC3108268

[ref83] Singh D. Shi L. Adams J. M. (2013). Bacterial diversity in the mountains of south-West China: climate dominates over soil parameters. J. Microbiol. 51, 439–447. doi: 10.1007/s12275-013-2446-9, PMID: 23990294

[ref84] Singh D. Takahashi K. Kim M. Chun J. Adams J. M. (2012). A hump-backed trend in bacterial diversity with elevation on Mount Fuji, Japan. Microb. Ecol. 63, 429–437. doi: 10.1007/s00248-011-9900-1, PMID: 21735154

[ref85] Song X. Cao M. Li J. Kitching R. L. Nakamura A. Laidlaw M. J.. (2021). Different environmental factors drive tree species diversity along elevation gradients in three climatic zones in Yunnan, southern China. Plant Divers. 43, 433–443. doi: 10.1016/j.pld.2021.04.006, PMID: 35024512 PMC8720829

[ref86] Sorensen P. O. Germino M. J. Feris K. P. (2013). Microbial community responses to 17 years of altered precipitation are seasonally dependent and coupled to co-varying effects of water content on vegetation and soil C. Soil Biol. Biochem. 64, 155–163. doi: 10.1016/j.soilbio.2013.04.014

[ref87] Sutherland W. J. Freckleton R. P. Godfray H. C. J. Beissinger S. R. Benton T. Cameron D. D.. (2013). Identification of 100 fundamental ecological questions. J. Ecol. 101, 58–67. doi: 10.1111/1365-2745.12025

[ref88] Tang Z. Fang J. Y. (2004). A review on the elevational patterns of plant species diversity. Biodivers. Sci. 12, 20–28. doi: 10.17520/biods.2004004

[ref89] Tedersoo L. Bahram M. Põlme S. Kõljalg U. Yorou N. S. Wijesundera R.. (2014). Global diversity and geography of soil fungi. Science 346:1078. doi: 10.1126/science.1256688 25430773

[ref90] Truu M. Ostonen I. Preem J. K. Lõhmus K. Nõlvak H. Ligi T.. (2017). Elevated air humidity changes soil bacterial community structure in the silver birch stand. Front. Microbiol. 8:557. doi: 10.3389/fmicb.2017.00557 28421053 PMC5376589

[ref91] Unger M. Leuschner C. Homeier J. (2010). Variability of indices of macronutrient availability in soils at different spatial scales along an elevation transect in tropical moist forests (NE Ecuador). Plant Soil 336, 443–458. doi: 10.1007/s11104-010-0494-z

[ref92] Wang J. Cao P. Hu H. Li J. Han L. L. Zhang L.. (2014). Altitudinal distribution patterns of soil bacterial and Archaeal communities along Mt. Shegyla on the Tibetan plateau. Microb. Ecol. 69, 135–145. doi: 10.1007/s00248-014-0465-7 25074792

[ref93] Wang J. Hu A. Meng F. Zhao W. Yang Y. Soininen J.. (2022). Embracing mountain microbiome and ecosystem functions under global change. New Phytol. 234, 1987–2002. doi: 10.1111/nph.18051, PMID: 35211983

[ref94] Wang J. Soininen J. Zhang Y. Wang B. Yang X. Shen J. (2011). Contrasting patterns in elevational diversity between microorganisms and macroorganisms. J. Biogeogr. 38, 595–603. doi: 10.1111/j.1365-2699.2010.02423.x

[ref95] White T. Bruns T. Lee S. Taylor J. Innis M. Gelfand D.. (1990). “Amplification and direct sequencing of fungal ribosomal RNA genes for Phylogenetics” in PCR Protocols. eds. Innis M. A. Gelfand D. H. Sninsky J. J. White T. J. (Cambridge: Academic Press), 315–322.

[ref96] Wickham H.. (2016). Ggplot2: Elegant Graphics for Data Analysis. Springer-Verlag New York.

[ref97] Xin F.. (2013). *Altitudinal Pattern of Plants Species Richness in Meili Snow Mountain and a Test of Rapoport's Rule*. Yunnan University (Master's Thesis). (In Chinese with English Abstract).

[ref98] Yang X. Baskin C. C. Baskin J. M. Pakeman R. J. Huang Z. Gao R.. (2021). Global patterns of potential future plant diversity hidden in soil seed banks. Nat. Commun. 12:7023. doi: 10.1038/s41467-021-27379-1, PMID: 34857747 PMC8639999

[ref99] Zhang J.. (2019). *Plantlist: Looking up the Status of Plant Scientific Names Based on the Plant List Database*. Available at: http://R-Forge.R-project.org.

[ref100] Zhang Y. Liu Y. Sun L. Baskin C. C. Baskin J. M. Cao M.. (2022). Seed dormancy in space and time: global distribution, paleoclimatic and present climatic drivers, and evolutionary adaptations. New Phytol. 234, 1770–1781. doi: 10.1111/nph.18099, PMID: 35292965

[ref101] Zhang Y. Z. Qian L. S. Chen X. F. Sun L. Sun H. Chen J. G. (2022). Diversity patterns of cushion plants on the Qinghai-Tibet plateau: a basic study for future conservation efforts on alpine ecosystems. Plant Divers. 44, 231–242. doi: 10.1016/j.pld.2021.09.001, PMID: 35769589 PMC9209862

[ref102] Zhang Y. Qian L. Spalink D. Sun L. Chen J. Sun H. (2021). Spatial phylogenetics of two topographic extremes of the Hengduan Mountains in southwestern China and its implications for biodiversity conservation. Plant Divers. 43, 181–191. doi: 10.1016/j.pld.2020.09.001, PMID: 34195502 PMC8233532

[ref103] Zhang R. Shen H. Dong S. Li S. Xiao J. Zhi Y.. (2022). Effects of 5-year nitrogen addition on species composition and diversity of an alpine steppe plant community on Qinghai-Tibetan plateau. Plan. Theory 11:966. doi: 10.3390/plants11070966, PMID: 35406946 PMC9002499

[ref104] Zheng Y. Chen L. Ji N. N. Wang Y. L. Gao C. Jin S. S.. (2021). Assembly processes lead to divergent soil fungal communities within and among 12 forest ecosystems along a latitudinal gradient. New Phytol. 231, 1183–1194. doi: 10.1111/nph.17457, PMID: 33982802

[ref105] Zhou J. Ning D. (2017). Stochastic community assembly: does it matter in microbial ecology? Microbiol. Mol. Biol. Rev. 81:17. doi: 10.1128/MMBR.00002-17, PMID: 29021219 PMC5706748

