# Supplementary material for: The spatial patterns of diversity and their relationships with environments in rhizosphere microorganisms and host plants differ along elevational gradients
Source: Front Microbiol. 2023 Feb 23;14:1079113. doi: 10.3389/fmicb.2023.1079113 (PMC9996296; doi:10.3389/fmicb.2023.1079113)
Supplement: Supplementary file 1 [file Data_Sheet_1.docx]

**Supplementary Information for**

**The spatial patterns of diversity and their relationships with environments in rhizosphere microorganisms and host plants differ along elevational gradients**

Shijia Xu^1,2^†, Yan Yuan^2^†, Pengfei Song^1,2^, Mufeng Cui^1,2^, Rensheng Zhao^1,2^, Xiaoyang Song^1^, Min Cao^1^, Yazhou Zhang^1,^*, Jie Yang^1,^*

* Yazhou Zhang, zhangyazhou@xtbg.ac.cn;

Jie Yang, yangjie@xtbg.org.cn

**Methods**

**Study sites.** Yunnan Province in southern China lies in the transitional region between the Hengduan Mountains and Asian tropics, where it borders Guizhou, Guangxi, Sichuan and Tibet in China and the countries of Myanmar, Laos and Vietnam [1]. Yunnan covers a total area of 394,000 km2 with terraced terrain and an average elevation of 2000 m [2]. It is mostly an uplifted, high elevation region with a terraced topography stretching from the northwest (maximum elevation at 6740 m) to the south (minimum at 76 m) [2]. The whole of Yunnan Province experiences a monsoonal climate, which leads to distinct rainy seasons (May-October) and dry seasons (November-April) [3]. Three major climatic zones are recognized in accordance with the three basal terrains: subalpine (ca. 3000 m), subtropical (ca. 2000 m) and tropical (ca. 600-800 m) [4]. Correspondingly, the vegetation in this province also comprises three regional types: montane coniferous forest on the Qinghai-Tibet Plateau; subtropical evergreen broad-leaved forest, and tropical forest [5]. The topographic configuration of Yunnan and its distinct dry and wet seasons are thought to have been critical to the development of biodiversity in this region, which is both a cradle and a museum of plant diversity [1]. Yunnan Province harbors fascinating biodiversity, e.g. ca. 18000 plant species [6], and thus this region is an ideal platform to study biodiversity [7].

**Molecular analyses.** PCR amplification conditions. The PCR components contained 5 μl of buffer (5×), 0.25 μl of Fast pfu DNA Polymerase (5U/μl), 2 μl (2.5 mM) of dNTPs, 1 μl (10 uM) of each Forward and Reverse primer, 1 μl (20 ng/μl) of DNA Template, and 14.75 μl of ddH2O. Thermal cycling consisted of initial denaturation at 98 °C for 5 min, followed by 25 cycles consisting of denaturation at 98 °C for 30 s, annealing at 53 °C for 30 s, and extension at 72 °C for 45 s, with a final extension of 5 min at 72 °C. PCR amplicons were purified with Vazyme VAHTSTM DNA Clean Beads (Vazyme, Nanjing, China) and quantified using the Quant-iT PicoGreen dsDNA Assay Kit (Invitrogen, Carlsbad, CA, USA). After the individual quantification step, amplicons were pooled in equal amounts, and pair-end 2×250 bp sequencing was performed using the Illumina NovaSeqplatform with NovaSeq 6000 SP Reagent Kit (500 cycles) at Shanghai Personal Biotechnology Co., Ltd (Shanghai, China).

**Soil property measurements**

**Table S1**. All measurements were followed the protocols of Public Technology Service Center, Xishuangbanna Tropical Botanical Garden, Chinese Academy of Sciences (http://english.xtbg.cas.cn/ptsc/pl). The test item, instruments and standard methods are as following:

| Sample types | Test item | Instruments | Standard methods |
| --- | --- | --- | --- |
| Soil | organic matter | Auto Kjeldahl Unit | (LY/T 1237-1999 )/Determination of organic matter and calculation of C/N ratio in forest soil |
|  | total carbon | Macro Elemental CN Analyzer | Macro Elemental CN Analyzer/Determination of total carbon in forest soil |
|  | total nitrogen | Macro Elemental CN Analyzer | Macro Elemental CN Analyzer/Determination of total nitrogen in forest soil |
|  | hydrolytic nitrogen | Auto Kjeldahl Unit | (LY/T 1229-1999)/Determination of hydrolyzable nitrogen in forest soil |
|  | total phosphorus | Atomic Absorption Spectrophotometer | (LY/T 1254-1999)/ Determ ination of total phosphorus in forest soil |
|  | total potassium | Atomic Absorption Spectrophotometer | (LY/T 1254-1999)/ Determ ination of total potassium in forest soil |
|  | available potassium | iCAP6300；IRIS Advantage | (LY/T 1236-1999 )/ Determination of available potassium in forest soil |
|  | pH | pH | (LY/T 1239-1999)/Determination of pH value in forest soil |
|  | water content | Double beam UV-Visible spectrophotometer | (LY/T 1213-1999)/Determination of forest soil water content |

**Table S2**. Mean values and standard deviations of environmental factors on different elevational gradients.

|  | temperature | humidity | organic matter | total carbon | total nitrogen | hydrolytic nitrogen | total phosphorus | total potassium | available potassium | pH | water |
| --- | --- | --- | --- | --- | --- | --- | --- | --- | --- | --- | --- |
|  |  |  |  |  |  |  |  |  |  |  |  |
| 800 | 20.09±0.41 | 94.88±1.64 | 20.21±3.19 | 12.78±1.93 | 1.44±0.10 | 404.00±66.72 | 0.33±0.02 | 11.23±1.22 | 198.92±59.63 | 4.94±0.28 | 0.29±0.05 |
| 1000 | 20.37±0.19 | 89.35±2.10 | 35.44±4.33 | 22.52±1.18 | 1.99±0.11 | 353.20±47.50 | 0.34±0.05 | 10.94±2.36 | 217.94±86.01 | 4.62±0.21 | 0.37±0.05 |
| 1200 | 19.73±0.39 | 87.36±1.52 | 39.92±5.31 | 25.24±3.29 | 1.82±0.26 | 155.80±24.29 | 0.22±0.03 | 7.16±0.75 | 162.51±37.96 | 4.42±0.06 | 0.40±0.04 |
| 1400 | 18.96±0.22 | 87.49±2.30 | 54.24±13.52 | 30.31±4.67 | 2.14±0.39 | 216.80±26.67 | 0.34±0.08 | 9.02±2.06 | 213.95±52.59 | 4.32±0.10 | 0.44±0.05 |
| 2000 | 14.29±0.11 | 87.38±2.00 | 91.09±12.73 | 57.31±7.68 | 4.22±0.53 | 161.60±21.21 | 0.79±0.18 | 25.60±8.90 | 108.80±31.61 | 4.80±0.15 | 0.55±0.04 |
| 2200 | 13.18±1.00 | 88.56±2.37 | 126.91±22.66 | 78.93±14.90 | 5.64±1.12 | 437.60±71.57 | 1.19±0.36 | 13.34±4.17 | 140.83±31.50 | 4.26±0.31 | 0.71±0.10 |
| 2400 | 12.25±0.16 | 91.82±1.60 | 144.69±10.43 | 91.45±3.63 | 6.21±0.36 | 362.60±53.88 | 1.07±0.17 | 21.74±7.98 | 94.71±20.46 | 4.50±0.17 | 0.84±0.25 |
| 2600 | 11.40±0.30 | 92.92±0.88 | 152.68±14.21 | 99.75±10.43 | 7.07±0.87 | 217.80±33.41 | 1.00±0.12 | 22.92±6.37 | 100.32±14.36 | 4.40±0.13 | 0.81±0.11 |
| 3200 | 7.67±0.14 | 79.00±1.73 | 136.35±33.89 | 83.28±21.09 | 7.18±1.84 | 223.49±49.74 | 1.04±0.22 | 12.63±1.81 | 80.72±24.75 | 5.67±0.62 | 0.58±0.07 |
| 3400 | 7.66±0.31 | 75.66±2.07 | 216.34±29.19 | 131.92±17.61 | 10.01±1.04 | 268.11±20.93 | 2.22±0.75 | 6.93±0.43 | 171.28±74.79 | 5.45±0.37 | 0.61±0.20 |
| 3600 | 6.19±0.28 | 77.08±1.67 | 151.39±21.93 | 89.15±13.09 | 6.98±1.62 | 191.73±40.92 | 1.83±0.27 | 7.90±0.25 | 93.19±30.17 | 5.45±0.10 | 0.52±0.08 |
| 3800 | 4.65±0.36 | 76.62±1.20 | 185.46±21.00 | 112.47±14.73 | 10.00±1.37 | 277.46±29.50 | 2.30±0.41 | 7.62±0.39 | 95.96±14.43 | 5.23±0.16 | 0.75±0.08 |

**Table S3**. For plant and microbial alpha diversity indexes in relation to elevation over various climatic zones, a comparison of AIC values from simple linear regression models (lm) and polynomial linear regression models (lm2).

| AIC | tropical | subtropical | subalpine |  | tropical | subtropical | subalpine |  | tropical | subtropical | subalpine |
| --- | --- | --- | --- | --- | --- | --- | --- | --- | --- | --- | --- |
|  | tree_SR | | |  | tree_PD | | |  | tree_Shan | | |
| lm2 | 115.61 | 109.47 | 84.71 |  | 285.47 | 263.34 | 277.07 |  | 16.11 | 12.12 | 13.08 |
| lm | 113.62 | 107.80 | 86.03 |  | 283.47 | 263.54 | 275.71 |  | 16.13 | 10.27 | 18.85 |
|  | fungal_SR | | |  | fungal_PD | | |  | fungal_Shan | | |
| lm2 | 340.00 | 323.84 | 301.71 |  | 261.71 | 253.62 | 246.02 |  | 6.57 | 14.60 | 31.53 |
| lm | 338.62 | 331.24 | 305.32 |  | 265.12 | 267.63 | 252.90 |  | 4.89 | 12.74 | 41.67 |
|  | bacteria_SR | | |  | bacteria_PD | | |  | bacteria_Shan | | |
| lm2 | 371.28 | 394.24 | 349.63 |  | 240.42 | 273.30 | 231.29 |  | -40.23 | -8.14 | -6.56 |
| lm | 379.29 | 394.58 | 356.40 |  | 260.15 | 272.26 | 236.69 |  | -9.52 | -9.88 | -2.12 |

**Table S4.** The following tables represent the individual explanatory rates of each variable for diversity.

| variable | plant_tropical(%) | plant_suntropical(%) | plant_subalpine(%) |
| --- | --- | --- | --- |
| A.K | 0.91 | 3.43 | 3.71 |
| altitude | 15.55 | 15.25 | 27.11 |
| humidity | 12.61 | 27.42 | 9.24 |
| Hy.N | 10.12 | 7.12 | 1.87 |
| O.M | 6.84 | 8.93 | 1.88 |
| pH | 8.41 | 2.25 | 1.51 |
| T.C | 8.05 | 8.08 | 2.59 |
| T.K | 5.62 | -1.65 | 10.69 |
| T.N | 4.53 | 8.1 | 6.13 |
| T.P | 5.25 | 3.24 | 8.02 |
| temperature | 16.79 | 15.19 | 23.75 |
| Water | 5.33 | 2.64 | 3.45 |
| Total_explained_variation | 59.1 | 36.4 | 81.4 |
|  |  |  |  |
|  |  |  |  |
|  |  |  |  |
|  |  |  |  |
|  |  |  |  |
|  |  |  |  |
|  |  |  |  |
|  | fungi_tropical(%) | fungi_suntropical(%) | fungi_subalpine(%) |
| A.K | 0.82 | 2.76 | 4.01 |
| altitude | 18.13 | 17.66 | 25.06 |
| humidity | 15.82 | 24.87 | 14.59 |
| Hy.N | 10.39 | 11.62 | 5.10 |
| O.M | 5.69 | 4.42 | 3.13 |
| pH | 4.16 | 5.16 | -0.47 |
| T.C | 6.77 | 5.20 | 3.05 |
| T.K | 3.84 | 1.13 | 6.17 |
| T.N | 4.32 | 6.48 | 5.75 |
| T.P | 6.61 | 2.52 | 4.22 |
| temperature | 20.73 | 16.41 | 26.11 |
| Water | 2.76 | 1.80 | 3.26 |
| Total_explained_variation | 80.30 | 71.50 | 76.30 |
|  |  |  |  |
|  |  |  |  |
|  |  |  |  |
|  |  |  |  |
|  |  |  |  |
|  |  |  |  |
|  |  |  |  |
|  | bacteria_tropical(%) | bacteria_suntropical(%) | bacteria_subalpine(%) |
| A.K | 0.57 | 3.29 | 4.36 |
| altitude | 17.39 | 17.53 | 24.12 |
| humidity | 16.5 | 24.15 | 13.52 |
| Hy.N | 10.07 | 11.73 | 4.61 |
| O.M | 6.22 | 4.05 | 3.15 |
| pH | 5.23 | 6.33 | 0.57 |
| T.C | 7.72 | 4.86 | 3.09 |
| T.K | 3.91 | 1.05 | 6.49 |
| T.N | 4.61 | 6.76 | 5.91 |
| T.P | 5.99 | 2.65 | 4.33 |
| temperature | 18.58 | 16.47 | 25.69 |
| Water | 3.28 | 1.21 | 4.18 |
| Total_explained_variation | 74.00 | 66.00 | 67.00 |

**Figure S1**

Relative abundances of the dominant microbial phyla in soils separated according to elevation gradients. Relative abundances are based on the proportional frequencies of those DNA sequences that could be classified at the phylum level. A: Fungi , B: Bacteria; SA: Subalpine, ST: Subtropical, TR: Tropical.


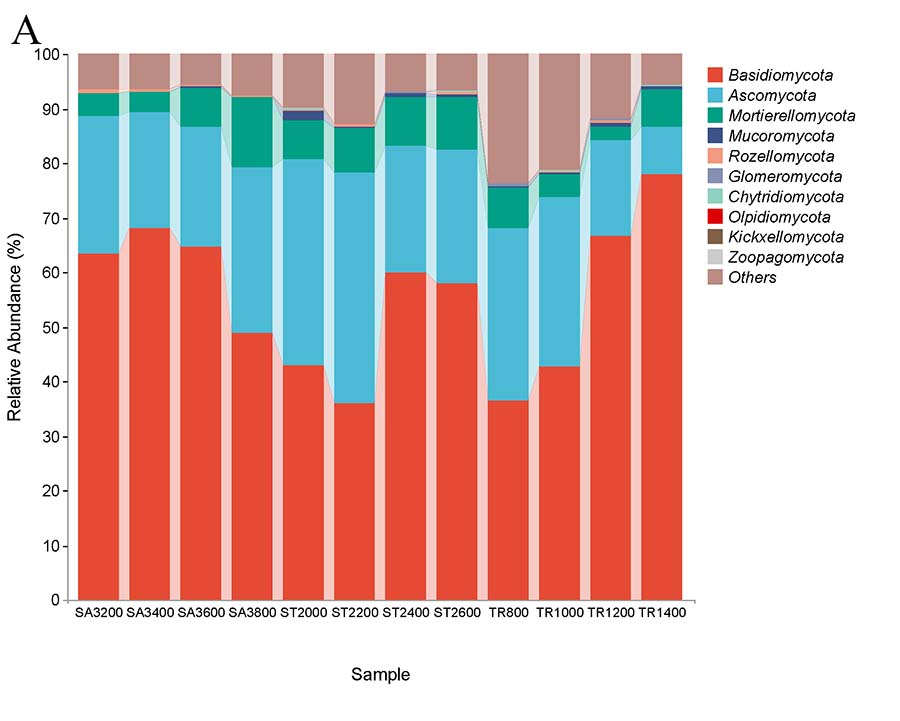

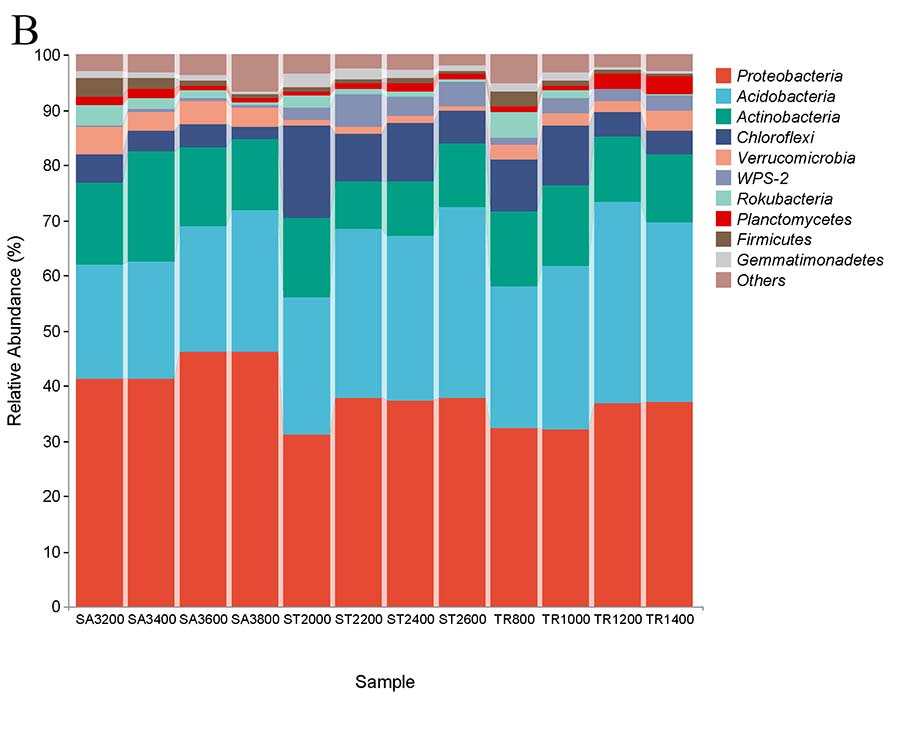


**Figure S2**

Changes in alpha diversity indexes of three fungal functional groups (Pathogen, Ectomycorrhiza and Arbuscular mycorrhiza) across the elevational gradients in three climatic zones. A: tropical; B: subtropical; C: subalpine; SR: species richness; PD: phylogenetic diversity; Shannon: Shannon‒Wiener index.


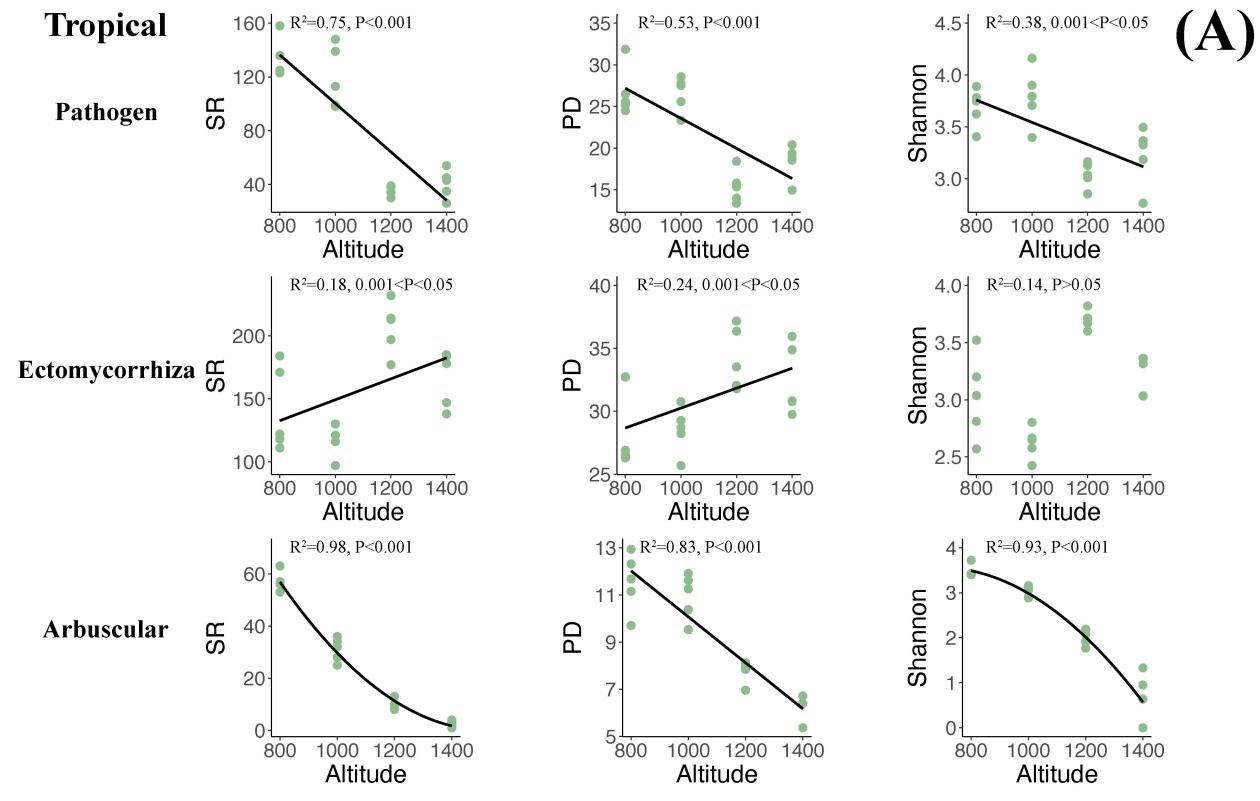


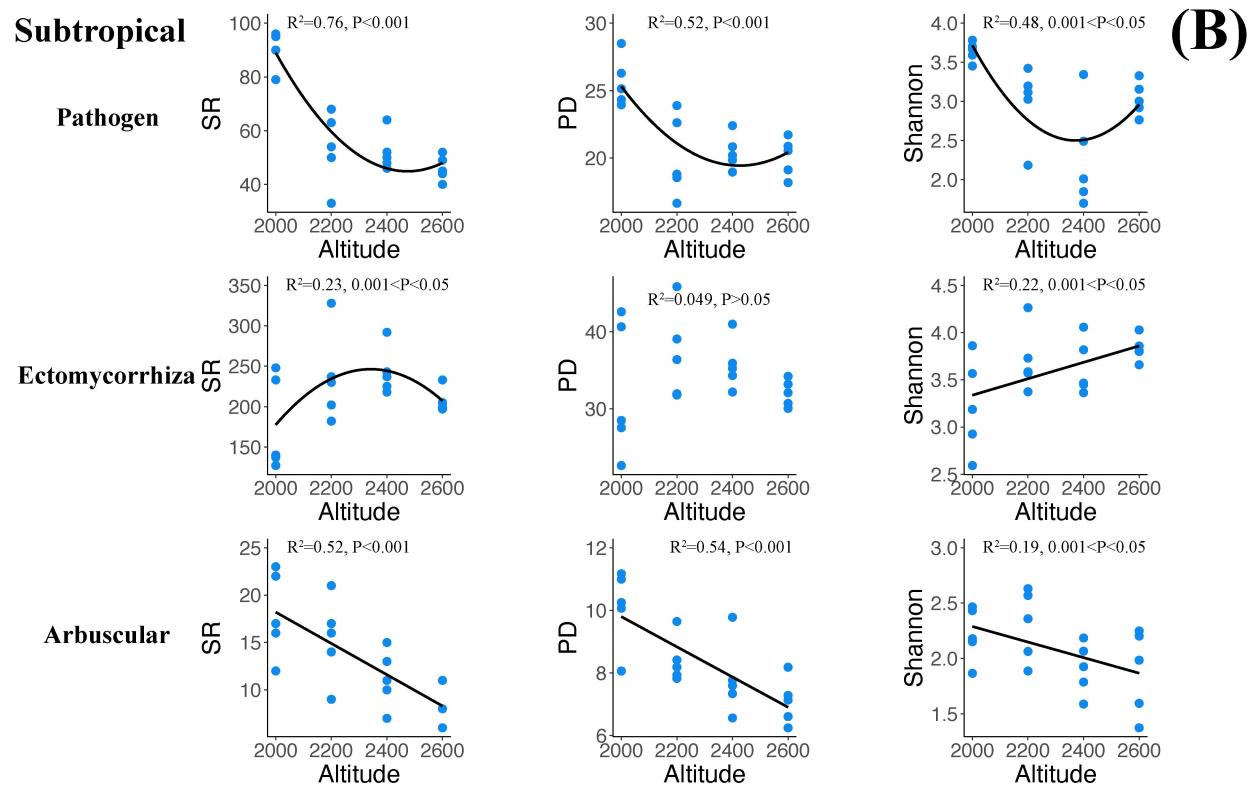


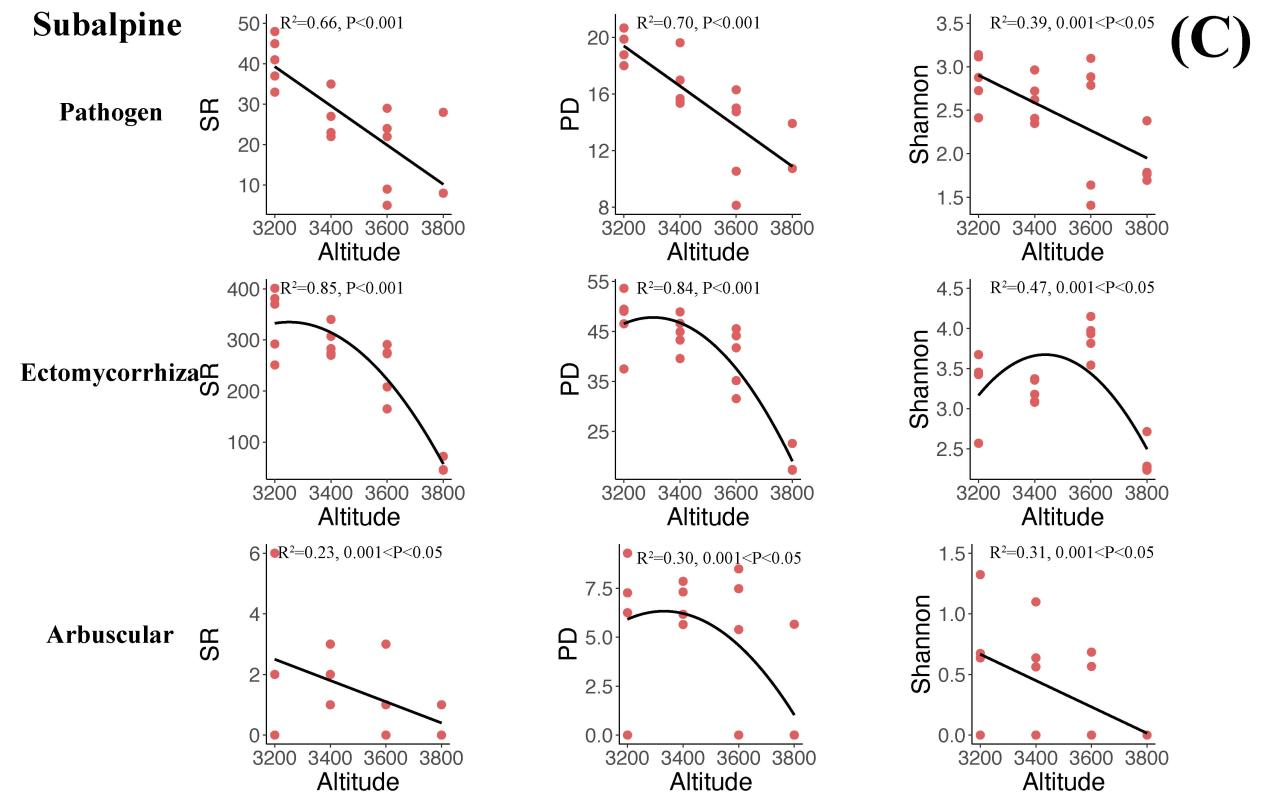


**Figure S3**

Changes in alpha diversity indexes of three bacterial functional groups (Nitrifier, Pathogen and nitrogen-fixing bacteria/N_fixation) across the elevational gradients in three climatic zones. A: tropical; B: subtropical; C: subalpine; SR: species richness; PD: phylogenetic diversity; Shannon: Shannon‒Wiener index.


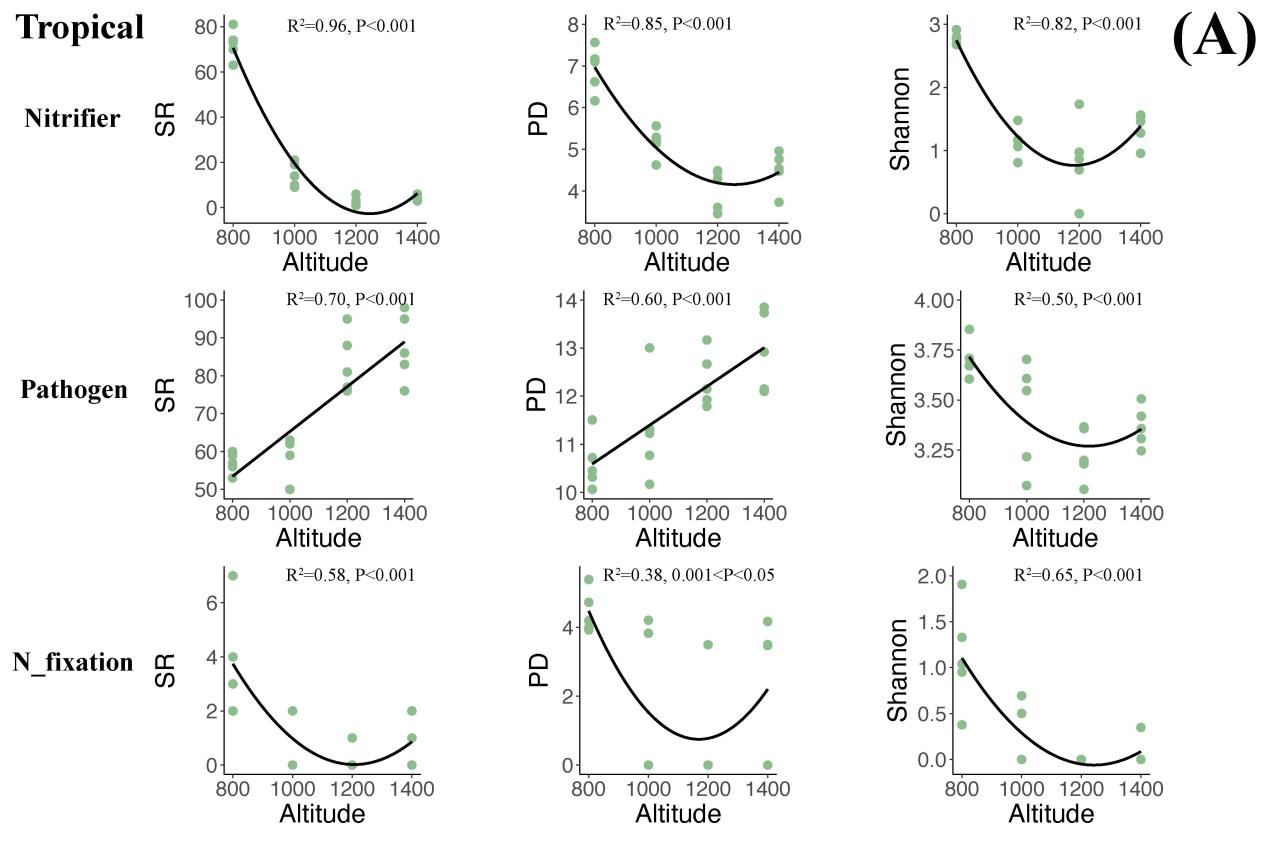


**
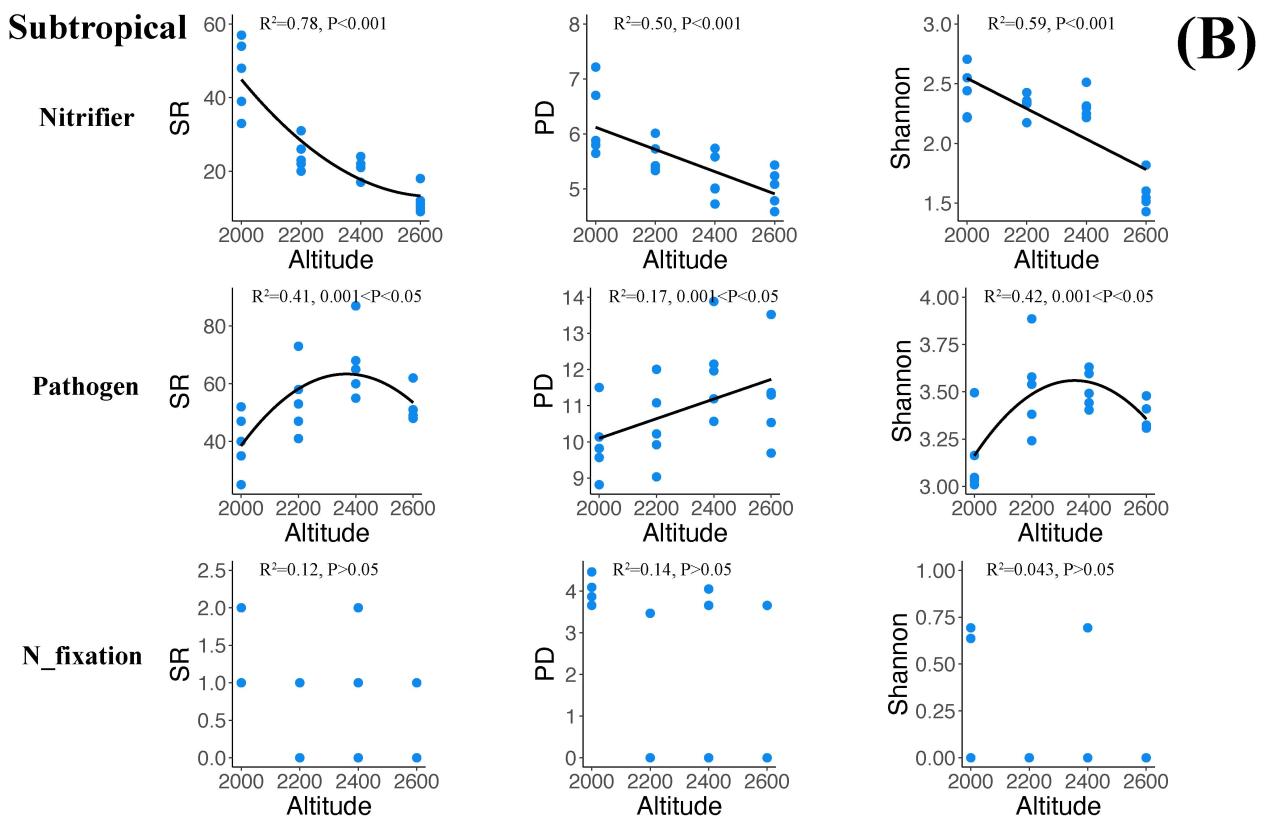
**

**
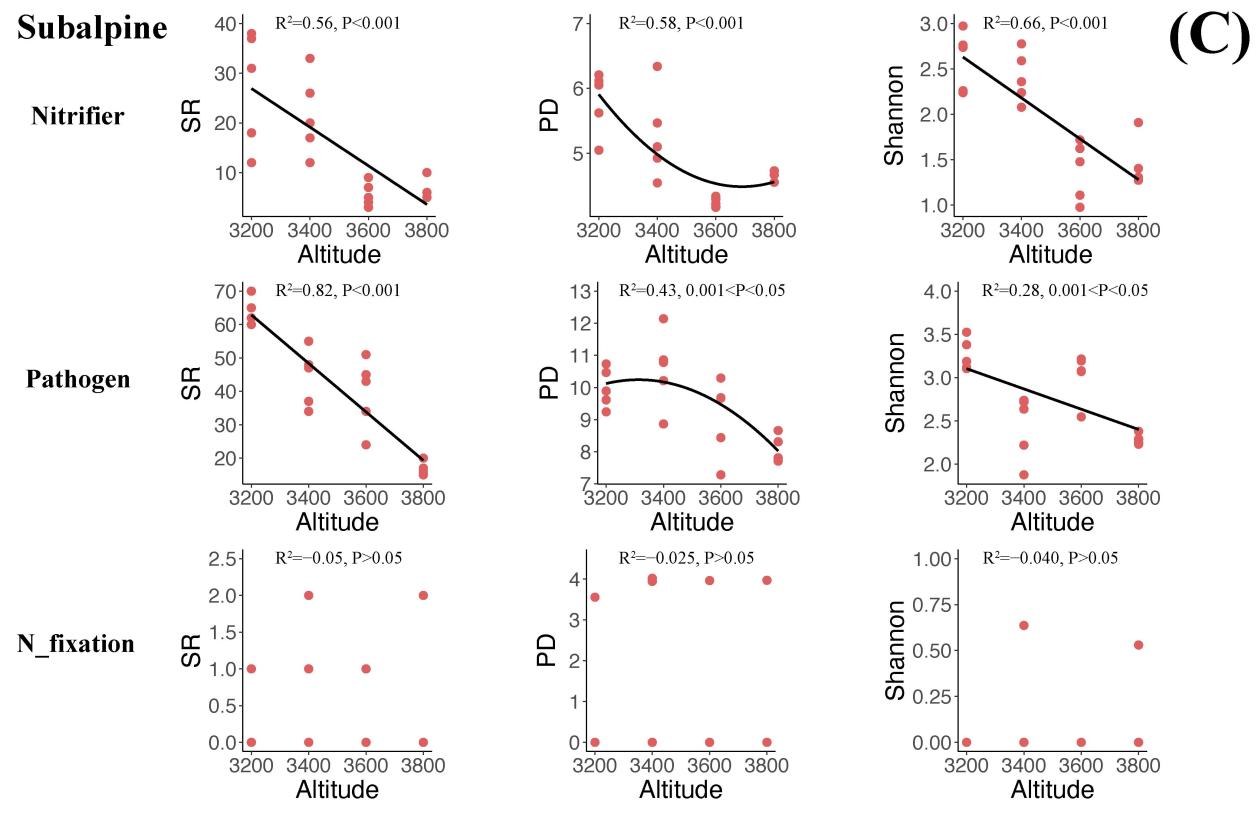
**

**Figure S4**

Relationships between soil factors and microbial functional groups diversity indexes in three climatic zones using random forest. A: Fungal functional groups (Pathogen, Ectomycorrhiza and Arbuscular mycorrhiza); B: Bacterial functional groups (Nitrifier, Pathogen and nitrogen-fixing bacteria/N_fixation); OM: soil organic matter content; TC: total carbon; TN: total nitrogen; HN: hydrolysable nitrogen; TP: total phosphorus; TK: total potassium; AK: available potassium; pH: soil pH; water: soil moisture content.

**
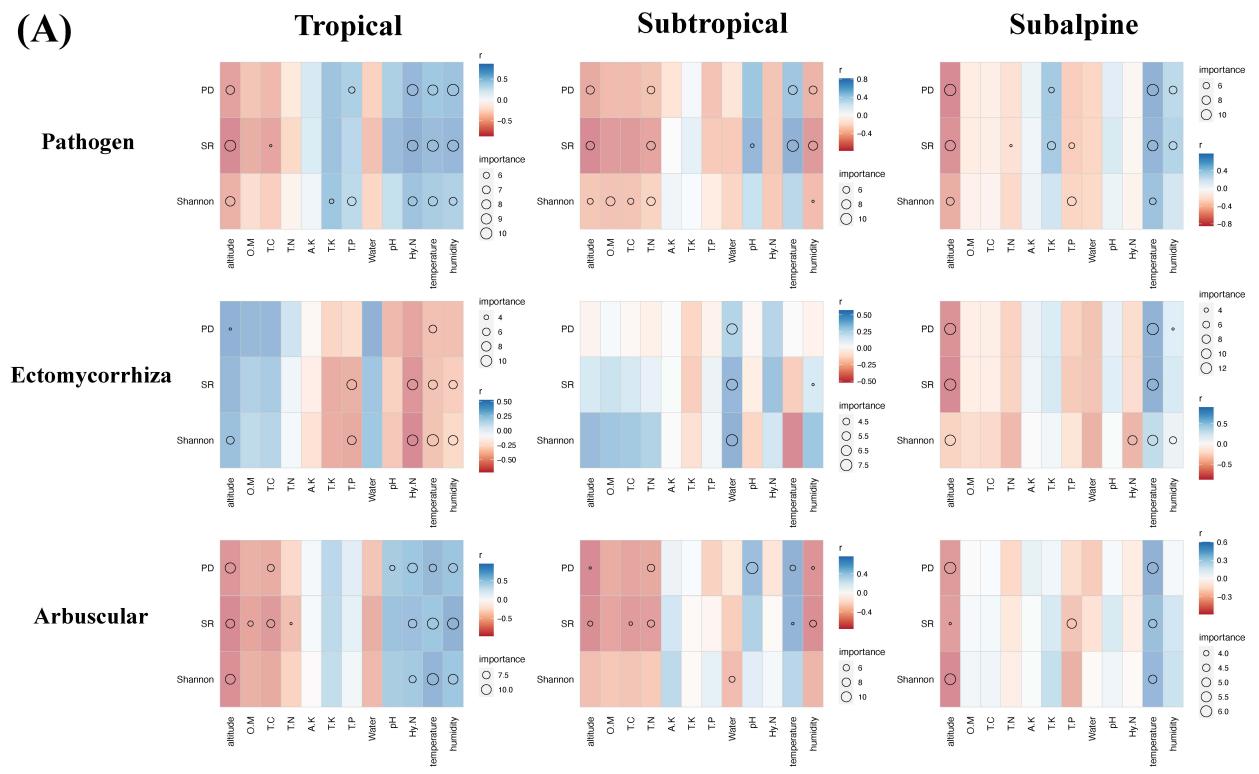
**

**
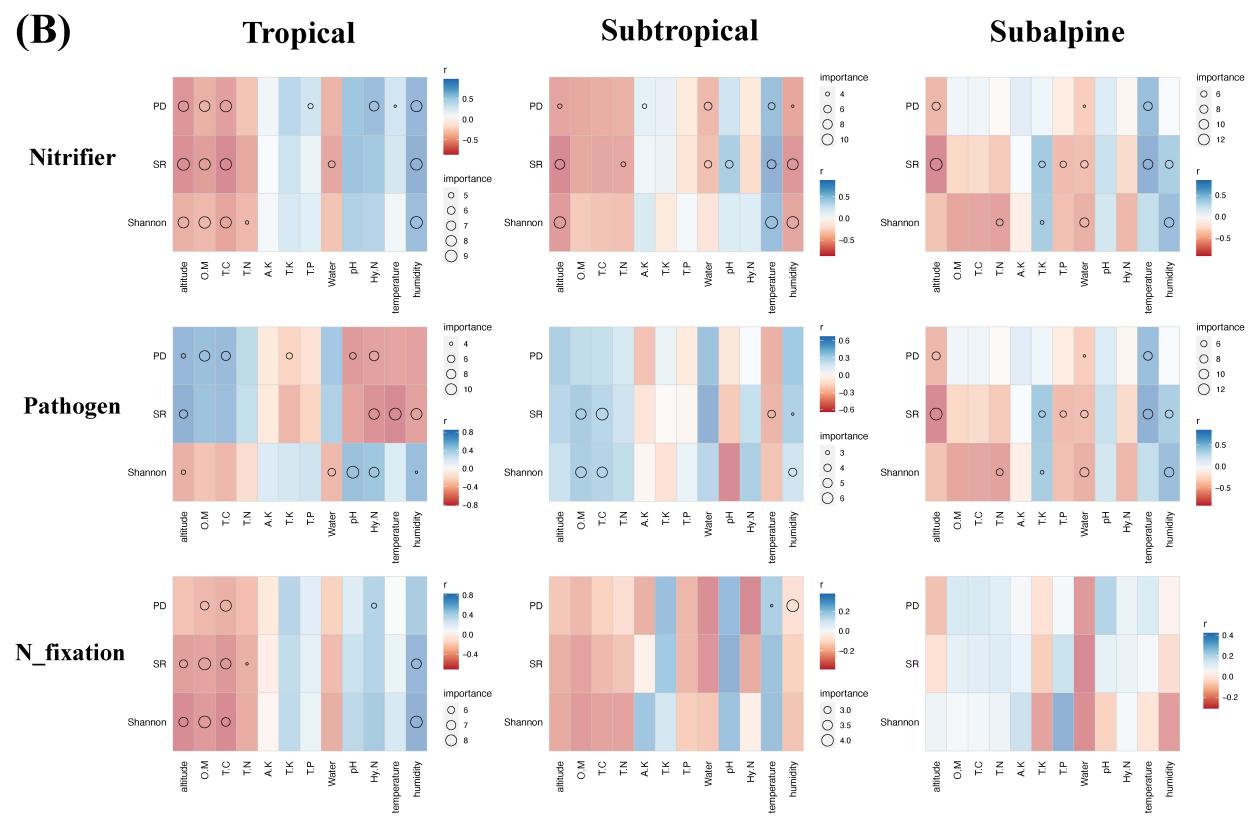
**

**Figure S5**

Nonmetric multidimensional scaling (NMDS) plots showing shifts in community composition for Plant (A), Rhizosphere fungi (B), and Rhizosphere bacteria (C) across tropical, subtropical and temperate climate zones. Solid lines indicate the two-dimensional space that contains all observations within a climate zone.


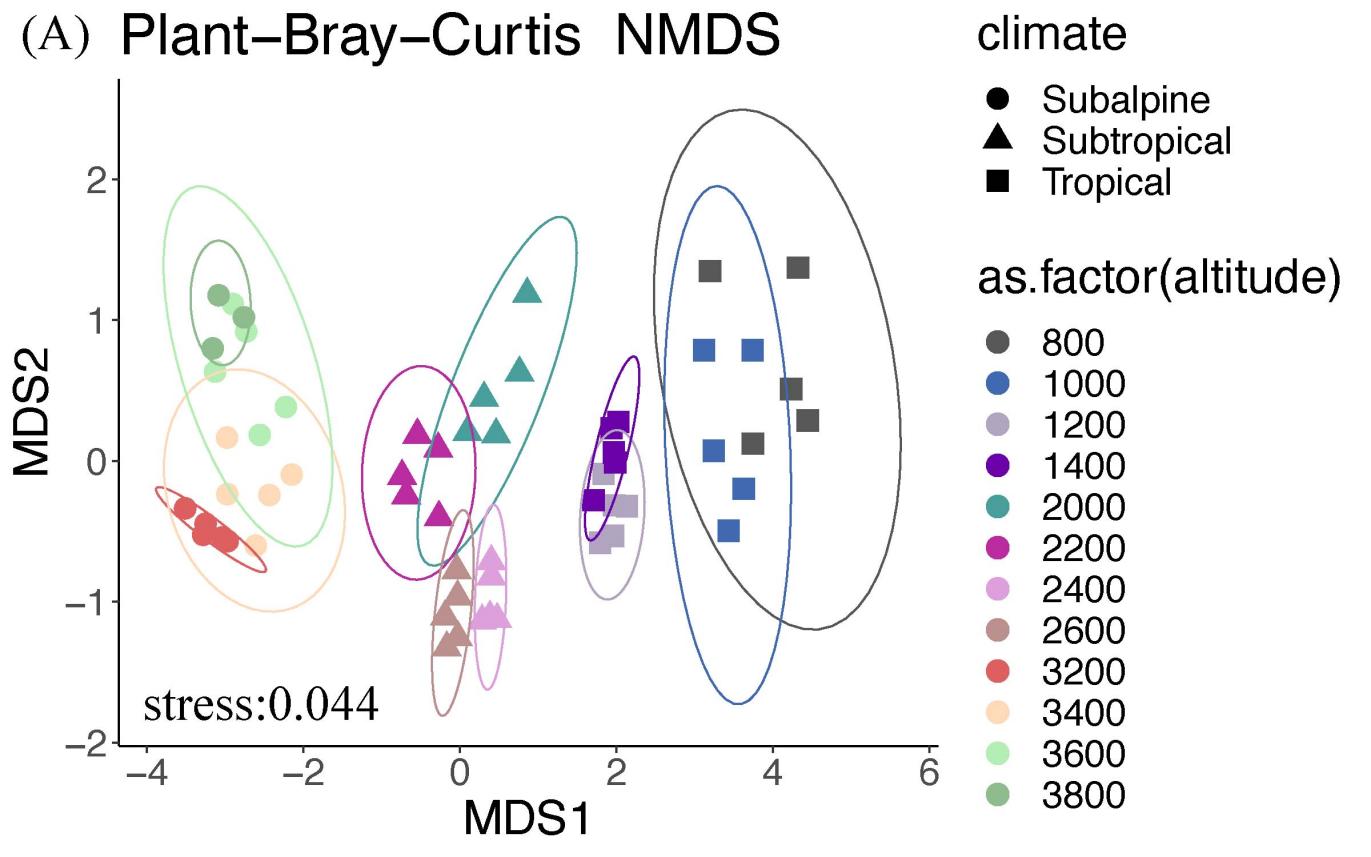


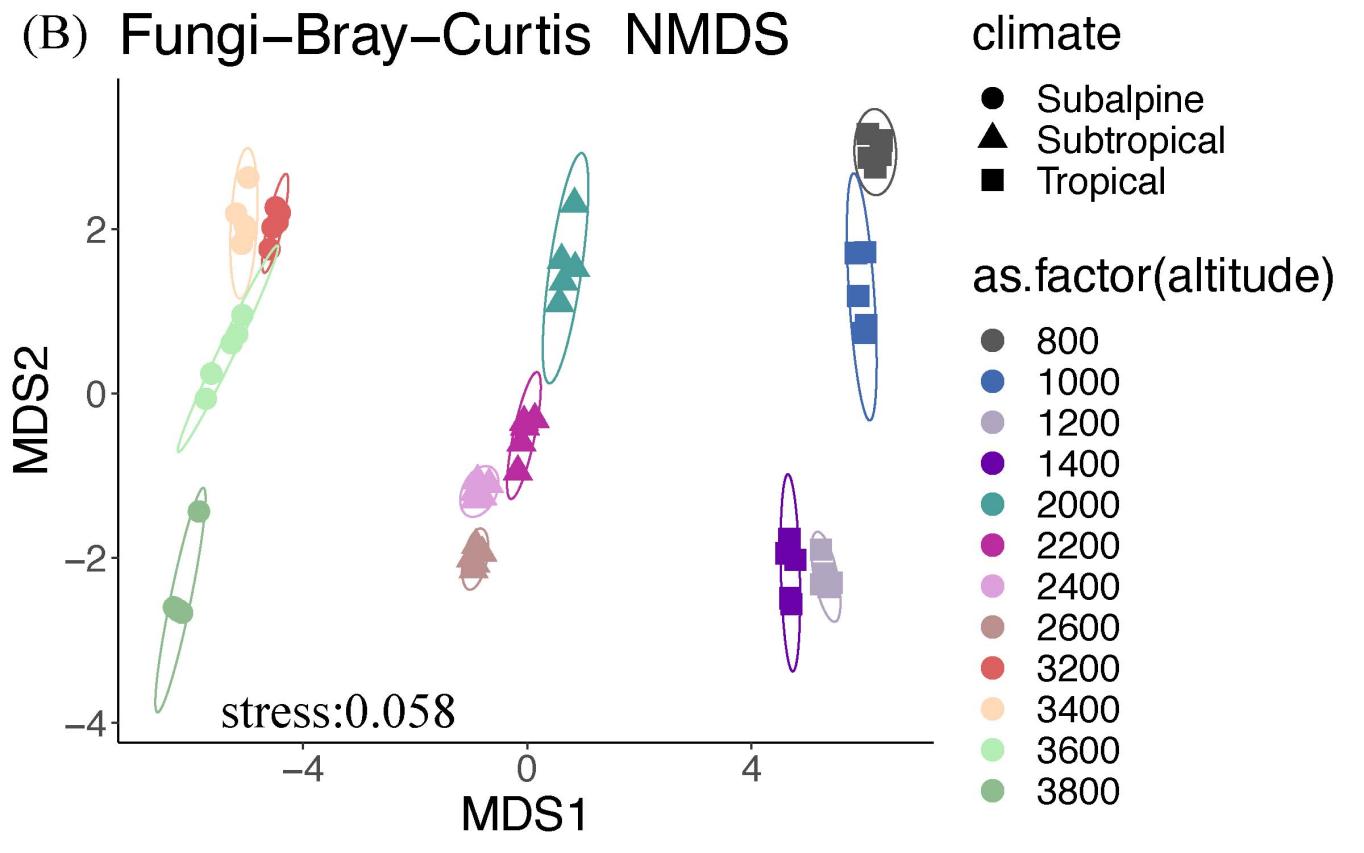


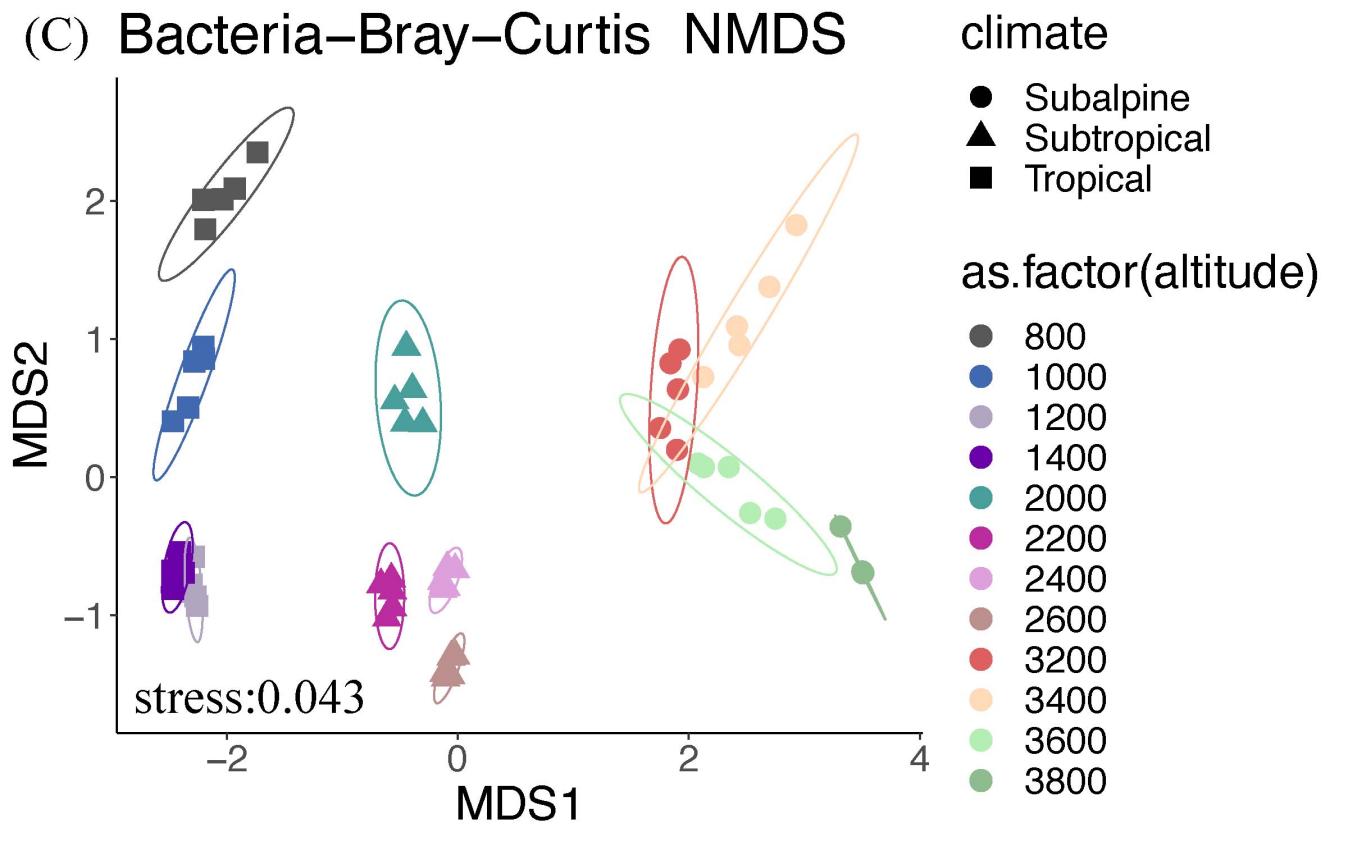


**References**

1. Qian L, Chen J-H, Deng T, Sun H (2020). Plant diversity in Yunnan: Current status and future directions. *Plant Divers.* **42:** 281-291.
2. Yang YM, Wang J, Wang JH (2008). *Studies on the Biodiversity and its Conservation in Yunnan, China*. Science Press: Beijing.
3. Song X, Cao M, Li J, Kitching RL, Nakamura A, Laidlaw MJ *et al* (2021). Different environmental factors drive tree species diversity along elevation gradients in three climatic zones in Yunnan, southern China. *Plant Divers.* **43:** 433-443.
4. Zhu H (2012). Biogeographical Divergence of the Flora of Yunnan, Southwestern China Initiated by the Uplift of Himalaya and Extrusion of Indochina Block. *PloS one* **7:** e45601.
5. Wu CY, Zhu YC, Jiang HQ (1987). *Vegetation of Yunnan*. Science Press: Beijing.
6. Kunming Institute of Botany Chinese Academy of Sciences (2006). *Flora of Yunnan*. Science Press: Beijing.
7. Li R, Kraft NJ, Yang J, Wang Y (2015). A phylogenetically informed delineation of floristic regions within a biodiversity hotspot in Yunnan, China. *Sci Rep* **5:** 9396.
8. Chen H (2021) VennDiagram: Generate High-Resolution Venn and Euler Plots. R package version 1.7.1. https://CRAN.R-project.org/package=VennDiagram.
